# OptimGS: a dual integrative genomic prediction framework for improving cold stress tolerance in wheat

**DOI:** 10.1093/bib/bbag375

**Published:** 2026-07-14

**Authors:** Prabina Kumar Meher, Farkhandah Jan, Nelofer Jan, Mukesh Rathore, Divya Sharma, Aanchal Gupta, Arzoo Kumari, Neeraj Budhlakoti, Sanjay Kalia, Amit Kumar Singh, Gyanendra Pratap Singh, Sundeep Kumar, Reyazul Rouf Mir

**Affiliations:** Division of Statistical Genetics, ICAR-Indian Agricultural Statistics Research Institute, Library Avenue, PUSA, New Delhi 110012, India; Division of Genetics and Plant Breeding, Faculty of Agriculture, Sher-e-Kashmir University of Agricultural Sciences and Technology of Kashmir, Wadura Campus, Sopore-193201, Kashmir, Jammu and Kashmir, India; Division of Genetics and Plant Breeding, Faculty of Agriculture, Sher-e-Kashmir University of Agricultural Sciences and Technology of Kashmir, Wadura Campus, Sopore-193201, Kashmir, Jammu and Kashmir, India; Division of Genetics and Plant Breeding, Faculty of Agriculture, Sher-e-Kashmir University of Agricultural Sciences and Technology of Kashmir, Wadura Campus, Sopore-193201, Kashmir, Jammu and Kashmir, India; Division of Genomic Resources, ICAR-National Bureau of Plant Genetic Resources, Library Avenue, PUSA, New Delhi 110012, India; Division of Statistical Genetics, ICAR-Indian Agricultural Statistics Research Institute, Library Avenue, PUSA, New Delhi 110012, India; Division of Statistical Genetics, ICAR-Indian Agricultural Statistics Research Institute, Library Avenue, PUSA, New Delhi 110012, India; Division of Agricultural Bioinformatics, ICAR-Indian Agricultural Statistics Research Institute, Library Avenue, PUSA, New Delhi 110012, India; Department of Biotechnology, Ministry of Science and Technology, CGO Complex, Lodhi Road, New Delhi 110003, India; Division of Genomic Resources, ICAR-National Bureau of Plant Genetic Resources, Library Avenue, PUSA, New Delhi 110012, India; Division of Genomic Resources, ICAR-National Bureau of Plant Genetic Resources, Library Avenue, PUSA, New Delhi 110012, India; Division of Genomic Resources, ICAR-National Bureau of Plant Genetic Resources, Library Avenue, PUSA, New Delhi 110012, India; Division of Genetics and Plant Breeding, Faculty of Agriculture, Sher-e-Kashmir University of Agricultural Sciences and Technology of Kashmir, Wadura Campus, Sopore-193201, Kashmir, Jammu and Kashmir, India; Centre for Crop and Food Innovation, WA State Agricultural Biotechnology Centre, Murdoch University, Murdoch, WA 6150, Australia

**Keywords:** genomic selection, ensemble strategy, cold stress tolerance, machine learning, chromosomal partition

## Abstract

Cold stress tolerance in wheat is a complex quantitative trait with low heritability, posing significant challenge for conventional breeding programs. Genomic selection offers a powerful framework for accelerating genetic gain; however, its prediction accuracy remains highly dependent on model choice and underlying genetic architecture. In this study, we propose a dual integrative genomic prediction framework designed to enhance prediction accuracy by sequentially integrating information across chromosomes and across models. Using a diverse wheat germplasm panel of 4269 genotypes evaluated for seedling cold tolerance over 2 years, we implemented 14 genomic prediction models spanning Bayesian, best linear unbiased prediction-based, and machine learning approaches. Genome-wide markers were first partitioned chromosome-wise, and predictions were generated independently for each chromosome. These predictions were then optimally combined using genetic algorithm under two bidirectional strategies: chromosome-first-model-second (CFMS) and model-first-chromosome-second (MFCS). Prediction performance was assessed through repeated five-fold cross-validation schemes, with Pearson’s correlation coefficient and mean squared error as performance metrics. The CFMS and MFCS strategies consistently outperformed individual models and conventional whole-genome approaches across all datasets. Overall, the proposed framework provides a robust and biologically meaningful strategy for improving genomic prediction of complex quantitative traits and holds potential for accelerating crop improvement programs. The source code of the developed framework is available at https://github.com/PrabinaMeher/OptimGS.git.

## Introduction

Wheat is one of the most essential cereal crops worldwide, accounting for ~37% of the average global cereal intake [1]. However, abiotic stresses associated with temperature fluctuations is one of the major threats for the production and productivity of this crop. Particularly, cold stress adversely affects the morphological, physiological, and biochemical processes of wheat, resulting in substantial yield losses. During the vegetative stage, cold stress delays seed germination, induces spikelet denaturation, and impairs root development [2, 3]. The reproductive stage is even more vulnerable, often leading to pollen sterility and incomplete grain set, resulting in a significant reduction in grain number [4–6]. Thus, there is a need to design breeding programs focused on developing cold-tolerant wheat varieties [7].

Breeding for cold tolerance using conventional methods depends upon phenotypic selection, which is both labor-intensive and time-consuming, often requiring 11–12 years to release a new variety [8]. Moreover, field-based phenotyping for winter hardiness, generally assessed by plant survivability, is not always reliable, as plants exhibit variable responses to winter severity [9, 10]. Phenotypic observations are also confounded by other abiotic and biotic stresses, adding further complexity in the selection process. Nonetheless, advent of next-generation sequencing technologies and low-cost genotyping platforms have revolutionized modern plant breeding programs by enabling early selection of plants based on genetic markers profile [single nucleotide polymorphism (SNP) markers]. Marker-assisted selection (MAS) is one such method which has been widely adopted for improving highly heritable traits [11–13]. However, the efficiency of MAS is limited when dealing with quantitative, low-heritable, and polygenic traits such as cold stress tolerance [13]. Genomic selection (GS) [14] is a modern breeding tool that offers promising alternative for breeding low heritable complex traits by incorporating genome-wide marker information.

The efficiency of GS is largely dependent upon genomic prediction (GP) accuracy, which measures the linear association between the genomic estimated breeding values (GEBVs) and observed phenotypic values. In other words, GP models estimate the effects of all markers across the genome and provide GEBVs, which is used to assess the genetic merit of an individual at an early stage of life for the target trait [14]. This enables breeders to identify superior genotypes earlier in the breeding cycle, accelerating genetic gain per unit time [15].

The accuracy of GP depends on both genetic and non-genetic factors, including heritability of the target trait, size and representativeness of the training population, marker density, proportion of markers in linkage disequilibrium with quantitative trait loci (QTL) and, most importantly, the statistical models used for prediction [16–18]. Traditionally, best linear unbiased prediction (BLUP) and its genomic counterpart, genomic BLUP (GBLUP), have served the foundation for GP, assuming normally distributed marker effects with equal variance, effective for highly polygenic traits [19, 20]. However, these models may underperform when marker effects vary greatly or when few loci exhibit large effects. To overcome these limitations, Bayesian models have been developed to allow varying markers effect size, better capturing the genetic architecture of complex traits [14, 21]. More recently, machine learning (ML) models such as random forest, extreme gradient boosting, and neural networks have been emerged as promising alternatives capable of capturing nonlinear relationships and epistasis interactions, often outperforming parametric models under complex genetic architectures [22–24].

While individual GP models have shown their potential to accelerate genetic gain, no single model consistently outperforms others across traits [25]. To overcome the limitations of individual-centric GP model, integration of predictions from multiple GP models have been reported as a promising strategy to enhance GP accuracy by leveraging the complementary strengths of diverse GP models [26]. Inspired by the diversity prediction theorem, Tomura et al. [26] developed an ensemble framework by averaging predicted phenotypes across different models. This ensemble approach achieved higher GP accuracy than the mean accuracy of individual models. Similarly, Chiaravallotti et al. [27] implemented an environment-ensemble strategy by training separate sub-models for each environment and then averaging their predictions. This method outperformed models trained on pooled multi-environment data, which emphasizes capability of ensemble modeling in capturing genotype × environment interactions. Several other studies have also reported the higher GP accuracy of ensemble approaches over individual GP models [28–34]. All these findings underscore the importance of ensemble model for achieving higher GP accuracy across diverse genetic architectures. In a nut shell, GP has become a cornerstone of modern breeding programs for both plant and animal species [16, 35, 36]. As far as cold stress tolerance in wheat is concerned, a few recent studies have explored the applicability of GS for winter survivorship and frost resistance in wheat [9, 37, 38]. GS has also been extended to indirect cold-survival traits, such as snow mold tolerance, with encouraging results [39, 40].

In this study, we developed a two-step integrative strategy to improve GP accuracy. First, genome-wide markers were partitioned by chromosome, and 14 GP models (Bayesian, BLUP, and ML) were applied independently to generate chromosome-level predictions. Genetic algorithm (GA) was then used to optimally weight and combine these chromosome-wise predictions for each model. In the second step, the model-specific integrated predictions were again combined using GA-derived weights to produce the final prediction. This hierarchical integration outperformed individual models and conventional whole-genome model integration, demonstrating its potential for enhancing GP across traits and crops.

## Materials and methods

### Phenotypic data

Screening of 4269 wheat genotypes for seedling cold tolerance was conducted under natural field conditions at Faculty of Agriculture (FoA), Wadura Campus, SKUAST-Kashmir, India. The experimental site is located at 33–37°N latitude and 72–80°E longitude, with an altitude of 1584 m above mean sea level. The experiment was conducted during 2020–21 and 2021–22 using an augmented block design. Each genotype was sown in a single row of 1 m length, with 20 cm spacing between rows and 10 cm between plants. The plots were maintained weed-, pest-, and disease-free, and standard agronomic practices were followed to ensure normal crop growth. Data were recorded on three randomly selected plants per genotype. Sowing was carried out in October for 2 consecutive years to expose the germplasm to natural cold stress during the winter months (November to March). Cold stress tolerance was assessed in the field using a 0–4 scale as described by Zhao et al. [41]: 0 (Highly tolerant): <10% of leaf area frozen; 1 (Tolerant): 11%–20% of leaf area frozen; 2 (Moderately tolerant): 21%–40% of leaf area frozen; 3 (Susceptible): 41%–70% of leaf area frozen; 4 (Highly susceptible): >71% of leaf area frozen. The original trait data set and more details about the data recording is available elsewhere [42]. Here, the ordinal scale 0–4 was used only to represent the degree of cold tolerance and not used as response variable in GP. In other words, the response variable used for GP was the proportion of leaf area frozen, which is quantitative in nature and lies between 0 and 1.

### Genotyping data

The genomic DNA from all 4269 lines was extracted separately from 15-day-old seedlings by following the Cetyltrimethylammonium bromide (CTAB) procedure [43]. Genotyping of the association panel was performed using a 35 K Axiom Wheat Breeders Array according to the method described by Affymetrix (Axiom 2. 0 Assay for 384 samples P/N 703154 Rev. 2) for wheat. All SNP markers with minimum allele frequency (MAF) < 0.1, maximum missing site per SNP > 20%, call rate < 90%, and not chromosomally annotated were excluded. The missing marker values were imputed using expectation–maximization algorithm available in rrBLUP R-package [44]. Finally, a total of 34 134 polymorphic SNPs were used for GP. Since wheat is a hexaploid species (2n = 6× = 42) composed of 3 subgenomes, namely A, B, and D, each containing 7 chromosomes, the wheat genome consists of a total of 21 chromosomes, represented as: 1A, 1B, 1D; 2A, 2B, 2D; …;7A, 7B, 7D. Here, the number 1–7 represents the homoeologous chromosome group, whereas the letters A, B, and D denote the 3 ancestral subgenomes of hexaploid wheat.

### Dataset for genomic prediction

We employed three datasets for GP, designated as D1, D2, and D3. The datasets D1 and D2 correspond to the experimental data collected during the years 2020–21 and 2021–22, respectively. The third dataset, D3, comprises the best linear unbiased estimates (BLUEs) obtained after adjusting for year effects using the following linear mixed-effects model:


$$ {Y}_{ij}=\mu +{G}_i+{E}_j+{GE}_{ij}+{\epsilon}_{ij} $$

where ${Y}_{ij}$ denotes the observed value of the trait corresponding to *i^th^* genotype ($i=1,2,\dots 4269$) in the *j^th^* environment ($j=1,2$), $\mu$ is the overall mean, ${G}_i$ is the fixed effect of the *i^th^* genotype, ${E}_j$ is the random effect of the *j^th^* environment (year), ${GE}_{ij}$ represents the random genotype–environment interaction effect, and ${\epsilon}_{ij}$ is the random residual term.

### Estimation of narrow sense heritability

The GBLUP mixed model was fitted for all the three datasets (D1, D2, and D3) independently. The GBLUP model can be written as $\boldsymbol{y}=\mu \mathbf{1}+\boldsymbol{Zg}+\boldsymbol{\epsilon}$**,** where $\boldsymbol{y}$ represents the numeric vector of observed phenotypic values for D1, D2, and vector of BLUEs for D3, $\mu$ is the overall mean, $\boldsymbol{g}$ is the vector of additive genetic effect and $\boldsymbol{g}\sim N\left(\mathbf{0},\boldsymbol{G}{\sigma}_g^2\right)$, $\boldsymbol{Z}$ is the design matrix for the additive genetic effect, $\boldsymbol{G}$ is the genomic relationship matrix computed using VanRaden method [20], $\boldsymbol{\epsilon}$ is the vector of residuals and $\boldsymbol{\epsilon} \sim N\left(\mathbf{0},\boldsymbol{I}{\sigma}_{\epsilon}^2\right)$. The GBLUP model was implemented using the rrBLUP R-package. After fitting of model using whole dataset, the narrow-sense heritability was estimated as ${h}^2=\frac{\sigma_g^2}{\sigma_g^2+{\sigma}_{\epsilon}^2}$, where ${\sigma}_g^2$ is the additive genetic variance and ${\sigma}_{\epsilon}^2$ is the residual variance.

### Genomic prediction model

In this study, we employed a total of 14 GP models comprising 8 Bayesian models, 2 BLUP-based models, and 4 ML models. The Bayesian models included BayesA [14], BayesB [14], BayesC [45], BayesBπ [14], BayesCπ [14], BayesR [46], BayesL [47], and BayesRR [35]. The BLUP-based approaches consisted of GBLUP [14] and rrBLUP [44]. The ML models included support vector machine (SVM), random forest (RF), extreme gradient boosting (XGB), and light gradient boosting machine (LGBM). The Bayesian models were implemented using the *hibayes* R package (version 1.0.0 available at https://cran.r-project.org/src/contrib/Archive/hibayes/) [48]. The Markov Chain Monte Carlo (MCMC) algorithm with parameters *nburn* = 14 000, *niter* = 20 000, and *thin* = 100 was employed for the Bayesian estimation. The *BGLR* R-package [21] was used for implementing GBLUP model, and *rrBLUP* R-package [44] was used for implementing the rrBLUP model. For ML models, *e1071* [49], *randomForest* [50], *xgboost* [51], and *lightgbm* [52] R-packages were utilized for implementing SVM, RF, XGB, and LGBM, respectively. A brief description of all the Bayesian, BLUP, and ML models is provided in the Supplementary File.

### Proposed integrated genomic prediction approach

All the genome-wide markers were partitioned chromosome-wise in $p$ sub sets ${C}_1,{C}_2,\dots, {C}_p$. For each chromosome ${C}_j$, $k$ prediction models ${M}_1$, ${M}_2$, …${M}_k$ were employed independently and vector of predicted trait values were obtained that is ${\hat{\boldsymbol{y}}}_{ij}\in{R}^n$, $i=1,2,..,k$; $j=1,2,\dots, p$. Here, ${\hat{\boldsymbol{y}}}_{ij}$=${\left({\hat{y}}_{ij}^{(1)},{\hat{y}}_{ij}^{(2)},\dots, {\hat{y}}_{ij}^{(n)}\right)}^{\prime }$and the observed phenotypic vector is $\boldsymbol{y}\in{R}^n$. In this study, we considered $k$ = 14 (comprising 8 Bayesian, 2 BLUP, and 4 ML models) and *P* = 21, corresponding to the 21 chromosomes of wheat. Two sequential bidirectional integration approaches were developed to combine predictions from multiple GP models and chromosomes. In the first approach, GP was performed independently for each chromosome, and the predicted phenotypic values for all the 21 chromosomes were integrated for each model followed by the integration of the integrated predicted values of all the models. In the second approach, the predicted phenotypic values of all the 14 models were integrated for each chromosome independently and then integration of the integrated phenotypic values of all chromosomes was done. We named the first approach as chromosome-first-model-second (CFMS) integration and the second approach as model-first-chromosome-second (MFCS) integration. Basically, both CFMS and MFCS approaches integrate predicted values across chromosomes and across GP models, but they differ in the order in which the integration was performed. In the CFMS approach, integration was first performed across chromosomes within each GP model, and then across models. In the MFCS approach, integration was first performed across models within each chromosome, followed by integration across chromosomes.

#### CFMS integration approach

The CFMS integration approach is explained below in simple steps.

- The whole marker dataset was divided chromosome-wise into 21 subsets corresponding to the wheat chromosomes (1A–7D). Each chromosome therefore represented a separate genomic feature set.
- For every chromosome, GP was performed separately using all the 14 models. This generated chromosome-wise predicted phenotypic values for every model. Thus, each model produced 21 chromosome-level predictions.
- For a given model, the predicted values obtained from all chromosomes were integrated using optimized chromosome weights obtained through GA. This step was repeated for all GP models separately. At the end of this step, each model produced one integrated prediction value.
- The integrated predictions obtained from different GP models were then combined using another round of GA-based weight optimization. This is the final integrated predicted phenotypic values.

The mathematical description for optimization of weight through GA and computation of the final integrated predicted phenotypic values are as follows.

For each model ${M}_i$, weighted sum of predicted values across chromosome was obtained as ${\hat{\boldsymbol{y}}}_{M_i}\left(\boldsymbol{w}\right)=\sum_{j=1}^p{w}_j{\hat{\boldsymbol{y}}}_{ij}$, here, $\boldsymbol{w}={\left({w}_1,{w}_2,\dots, {w}_p\right)}^{\prime }$ with constraints ${w}_j\ge 0$ and ${\sum}_{j=1}^p{w}_j=1$. The optimized value of $\boldsymbol{w}$ for each model ${M}_i$ was obtained by maximizing the objective function $f\left(\boldsymbol{w}\right)$ which is Pearson correlation coefficient (PCC) $r$ between the integrated predicted values ${\hat{\boldsymbol{y}}}_{M_i}\left(\boldsymbol{w}\right)$ and observed values $\boldsymbol{y}$ that is ${\boldsymbol{w}}^{(i)\ast }=\arg \underset{\mathbf{w}\in \mathbf{W}}{\max\ }f\left(\boldsymbol{w}\right)$, where $f\left(\boldsymbol{w}\right)$=$r\left(\boldsymbol{y},{\hat{\boldsymbol{y}}}_{M_i}\left(\boldsymbol{w}\right)\right)$, $\boldsymbol{W}=\left\{\boldsymbol{w}\boldsymbol{\in }{R}^p:{w}_{\boldsymbol{j}}\boldsymbol{\ge}0,{\sum}_{j=1}^p{w}_j=1\ \right\}$, and $r\left(\boldsymbol{y},{\hat{\boldsymbol{y}}}_{M_i}\left(\boldsymbol{w}\right)\right)=\frac{\mathit{\operatorname{cov}}\left(\boldsymbol{y},{\hat{\boldsymbol{y}}}_{M_i}\left(\boldsymbol{w}\right)\right)}{\sqrt{\mathit{\operatorname{var}}\left(\boldsymbol{y}\right)}\sqrt{\mathit{\operatorname{var}}\left({\hat{\boldsymbol{y}}}_{M_i}\left(\boldsymbol{w}\right)\right)}}$. After optimization, the integrated predicted values obtained for each model was ${\hat{\boldsymbol{y}}}_{M_i}^{\ast }$=${\hat{\boldsymbol{y}}}_{M_i}\left({\boldsymbol{w}}^{\left(\boldsymbol{i}\right)\ast}\right)$. The final predicted trait values were obtained by integrating integrated predicted values ${\hat{\boldsymbol{y}}}_{M_1}^{\ast },{\hat{\boldsymbol{y}}}_{M_2}^{\ast },\dots, {\hat{\boldsymbol{y}}}_{M_k}^{\ast }$ of the $k$ model using the weight $\boldsymbol{\mu} ={\left({\mu}_1,{\mu}_1,\dots, {\mu}_k\right)}^{\prime }$ with ${\mu}_{\boldsymbol{i}}\boldsymbol{\ge}0,{\sum}_{i=1}^k{\mu}_i=1$ that is ${\hat{\boldsymbol{y}}}_{CFMS}\left(\boldsymbol{\mu} \right)=\sum_{i=1}^k{\mu}_j{\hat{\boldsymbol{y}}}_{M_i}^{\ast }.$ The optimized values of $\boldsymbol{\mu}$ were obtained by maximizing the objective function ${f}_1\left(\boldsymbol{\mu} \right)$ which is the PCC ($r$) between the integrated predicted values ${\hat{\boldsymbol{y}}}_{CFMS}\left(\boldsymbol{\mu} \right)$ and observed values $\boldsymbol{y}$ that is ${\boldsymbol{\mu}}^{\ast }=\arg \underset{\boldsymbol{\mathrm{\mu}} \in \mathbf{M}}{\max\ }{f}_1\left(\boldsymbol{\mu} \right)$, where ${f}_1\left(\boldsymbol{\mu} \right)=r\left(\boldsymbol{y},{\hat{\boldsymbol{y}}}_{CFMS}\left(\boldsymbol{\mu} \right)\right)$, $\boldsymbol{M}=\left\{\boldsymbol{\mu} \boldsymbol{\in}{R}^k:{\mu}_{\boldsymbol{i}}\boldsymbol{\ge}0,{\sum}_{i=1}^k{\mu}_i=1\ \right\}$. The final predicted trait values were obtained as ${\hat{\boldsymbol{y}}}_{CFMS}={\hat{\boldsymbol{y}}}_{CFMS}\left({\boldsymbol{\mu}}^{\ast}\right)$.

#### MFCS integration approach

The MFCS integration framework is explained below in simple steps.

- Similar to CFMS, the marker dataset was first partitioned into chromosome-specific subsets.
- Each chromosome was analyzed separately using all 14 models.
- For a particular chromosome, predictions from 14 models were integrated using optimized model weights generated by the GA. This generated one integrated prediction per chromosome.
- The chromosome-level integrated predictions were then combined across all chromosomes using GA-optimized chromosome weights. This is the final integrated predicted phenotypic values in MFCS strategy.

The mathematical description for optimization of weight through GA and computation of the final integrated predicted phenotypic values are as follows.

For each chromosome ${C}_j$, weighted sum of predicted values of models was obtained as ${\hat{\boldsymbol{y}}}_{C_j}\left(\boldsymbol{\mu} \right)=\sum_{i=1}^k{\mu}_i{\hat{\boldsymbol{y}}}_{ij}$, where $\boldsymbol{\mu} ={\left({\mu}_1,{\mu}_1,\dots, {\mu}_k\right)}^{\prime }$ with constraints ${\mu}_{\boldsymbol{i}}\boldsymbol{\ge}0,{\sum}_{i=1}^k{\mu}_i=1$. The optimized value of $\boldsymbol{\mu}$ for each model ${C}_j$ was obtained by maximizing the objective function ${f}_2\left(\boldsymbol{\mu} \right)$ which the PCC ($r$) between the integrated predicted values ${\hat{\boldsymbol{y}}}_{C_j}\left(\boldsymbol{\mu} \right)$ and observed values $\boldsymbol{y}$ that is ${\boldsymbol{\mu}}^{(j)\ast }=\arg \underset{\boldsymbol{\mu} \in \mathbf{M}}{\max\ }{f}_2\left(\boldsymbol{\mu} \right)$, where ${f}_2\left(\boldsymbol{\mu} \right)=r\left(\boldsymbol{y},{\hat{\boldsymbol{y}}}_{C_j}\left(\boldsymbol{\mu} \right)\right)$, $\boldsymbol{M}=\left\{\boldsymbol{\mu} \boldsymbol{\in}{R}^k:{\mu}_{\boldsymbol{i}}\boldsymbol{\ge}0,{\sum}_{i=1}^k{\mu}_i=1\ \right\}$. After optimization, the integrated predicted values obtained for each chromosome was ${\hat{\boldsymbol{y}}}_{C_j}^{\ast }$=${\hat{\boldsymbol{y}}}_{C_j}\left({\boldsymbol{\mu}}^{(j)\ast}\right)$. The final predicted values were obtained by integrating the $p$ chromosome-level integrated predicted values ${\hat{\boldsymbol{y}}}_{C_1}^{\ast },{\hat{\boldsymbol{y}}}_{C_2}^{\ast },\dots, {\hat{\boldsymbol{y}}}_{C_p}^{\ast }$ using the weight $\boldsymbol{w}={\left({w}_1,{w}_2,\dots, {w}_p\right)}^{\prime }$ with ${w}_j\boldsymbol{\ge}0,{\sum}_{j=1}^p{w}_j=1$ that is ${\hat{\boldsymbol{y}}}_{MFCS}\left(\boldsymbol{w}\right)=\sum_{j=1}^p{w}_j{\hat{\boldsymbol{y}}}_{C_j}^{\ast }$. The optimized values of $\boldsymbol{w}$ were obtained by maximizing the objective function ${f}_1\left(\boldsymbol{w}\right)$ which is the PCC ($r\Big)$ between the integrated predicted values ${\hat{\boldsymbol{y}}}_{MFCS}\left(\boldsymbol{w}\right)$ and observed values $\boldsymbol{y}$ that is ${\boldsymbol{w}}^{\ast }=\arg \underset{\boldsymbol{w}\in \mathbf{W}}{\max\ }{f}_1\left(\boldsymbol{w}\right)$ where ${f}_1\left(\boldsymbol{w}\right)=r\left(\boldsymbol{y},{\hat{\boldsymbol{y}}}_{MFCS}\left(\boldsymbol{w}\right)\right)$, $\boldsymbol{W}=\left\{\boldsymbol{w}\boldsymbol{\in }{R}^p:{w}_{\boldsymbol{j}}\boldsymbol{\ge}0,{\sum}_{j=1}^p{w}_j=1\ \right\}$, and the final predicted trait values were obtained as ${\hat{\boldsymbol{y}}}_{MFCS}={\hat{\boldsymbol{y}}}_{MFCS}\left({\boldsymbol{w}}^{\ast}\right)$.

**Figure 1:**
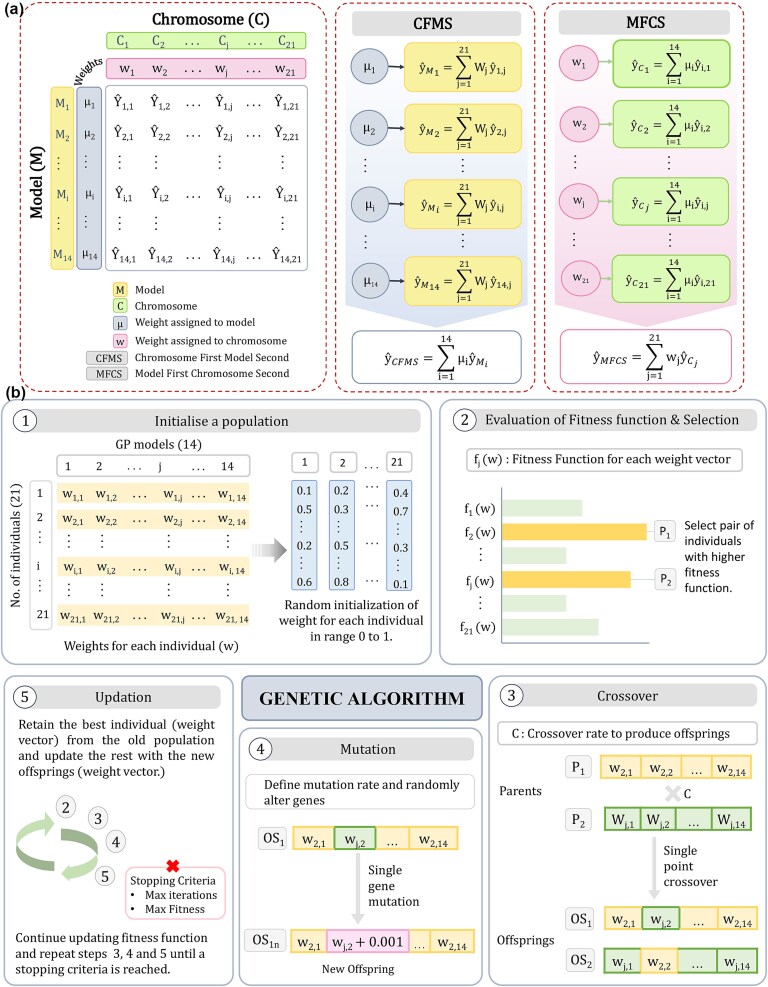
Schematic representation of the proposed integrated genomic prediction framework. (a) Illustration of the CFMS and MFCS approaches. (b) Workflow of the GA used to optimize model and chromosome weights.

The optimal weights ($\boldsymbol{\mu}, \boldsymbol{w}$) were estimated using GA optimization approach [53]. The GA was implemented using the *GA* R-package [54], with parameters initial population size (*m*) = 300, crossover rate = 0.2, mutation rate = 0.1, and maximum of 1000 iterations. The above combination of parameters for GA was adopted from the study of Meher et al. [25]. Utilizing 18 datasets differing in population size, marker density, and trait architecture, Meher et al. (2025) systematically evaluated the influence of different GA hyperparameter combinations on prediction accuracy and reported that above combination of parameters was most effective in convergence of fitness function and achieving higher GP accuracy. A schematic diagram of the CFMS and MFCS integration approaches is shown in Fig. 1a and the steps involved in the GA procedure are illustrated in Fig. 1b.

### Evaluation of genomic prediction accuracy with cross-validation

**Figure 2:**
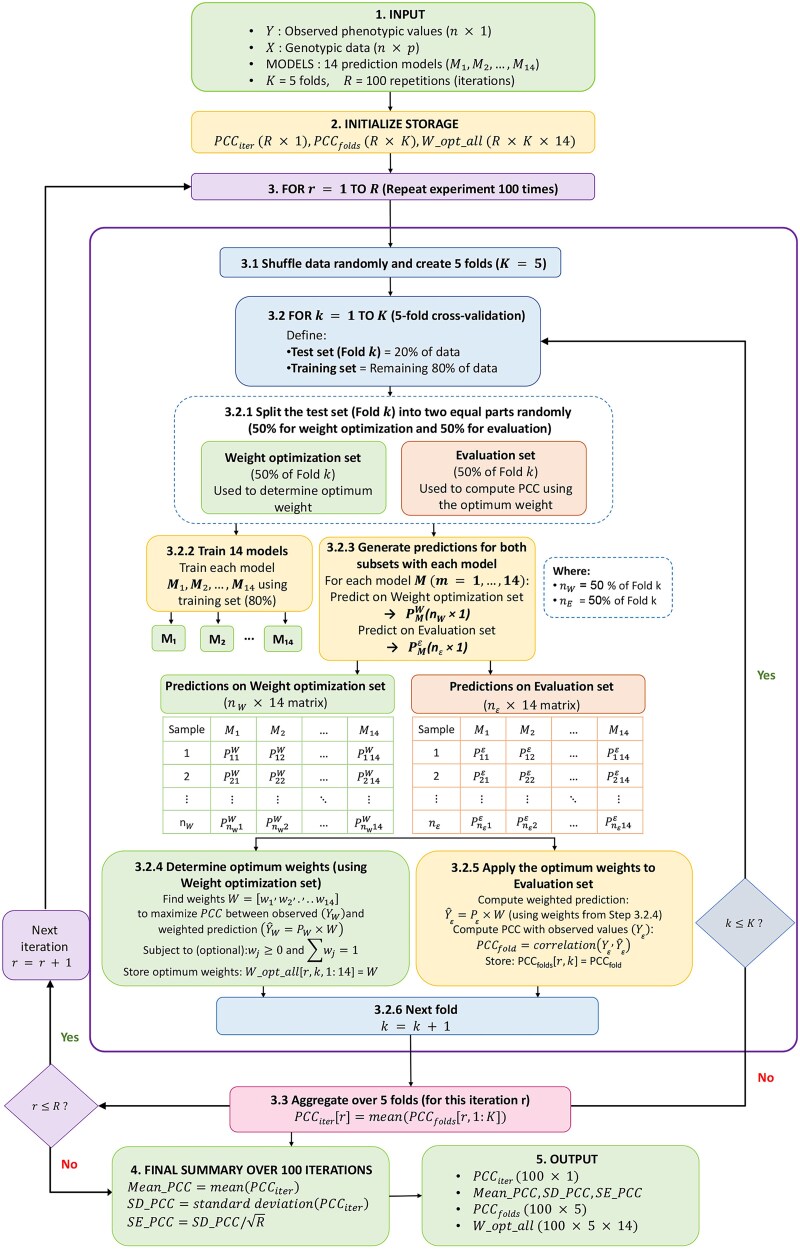
Workflow illustrating the GA-based optimization of weights at model level-ensemble framework. In each fold of the cross-validation, the test was randomly divided into two equal subsets: a weight optimization set and an evaluation set. The 14 genomic prediction models were trained using the training data, and predictions were generated separately for both subsets. The GA-based optimization procedure was then applied exclusively to the weight optimization subset to estimate the optimum ensemble weights by maximizing the PCC between observed and weighted predicted values. The optimized weights were subsequently applied to the independent evaluation subset to obtain integrated predictions and compute prediction accuracy. This procedure was repeated independently across all folds and iterations. Final prediction accuracy was estimated as the mean PCC across 5 folds and 100 repeated iterations.

A repeated five-fold cross-validation strategy was employed [55] for evaluating the performance of GP models. For five-fold cross-validation, the dataset was randomly divided into five equal subsets, with four subsets (80% of the data) used for model training and the remaining subset (20%) for testing in each fold, ensuring every subset served as a test set once. This process was repeated 5 times per iteration and iterated 100 times to minimize variability from random splits, resulting in robust accuracy estimates [56]. The GA-based weight optimization was fully nested within each fold of cross-validation iteration. Specifically, the weights were independently re-estimated for every fold and every repetition of the cross-validation process. In each fold of the cross-validation, only a randomly selected subset comprising 50% of the predicted phenotypic values of the test set was used to determine the optimum weights for models/chromosomes. These optimized weights were then applied to obtain weighted ensemble predictions using the remaining 50% of the test set of that fold. The same procedure was repeated independently for each fold and across all repetitions. After obtaining predictions for each fold, PCC and mean square error (MSE) were computed between observed ($y$) and integrated predicted (${\hat{y}}$) trait values to quantify the GP accuracy of the corresponding test set. The final prediction accuracy for each repetition was computed as the average across the 5 folds, and the overall accuracy was obtained by averaging across all 100 repetitions. Figure 2 depicted a flow diagram involving the steps from determination of weight to computation of prediction accuracy for ensemble of models. The PCC and MSE were computed using the following formula:


$$ PCC(r)=\frac{\sum_{i=1}^n\left({y}_i-\overline{y}\right)\left({{\hat{y}}}_i-\overline{\hat{y}}\right)}{\sqrt{\sum_{i=1}^n{\left({y}_i-\overline{y}\right)}^2{\sum}_{i=1}^n{\left({{\hat{y}}}_i-\overline{\hat{y}}\right)}^2}} $$

$$ MSE=\frac{1}{n}\sum_{i=1}^n{\left({y}_i-{{\hat{y}}}_i\right)}^2 $$

### Comparison with existing ensemble strategies

We compared the proposed ensemble strategy with different ensemble approaches reported in existing GP studies. For instance, Kick and Washburn [32] employed uniform weighting and inverse-variance weighting schemes to combine predictions from BLUP, ML, and deep learning models. Similarly, meta-learning or stacking-based ensemble approaches have also been explored for improving GP accuracy in earlier studies [57–59]. We performed a systematic comparison of the proposed framework with three ensemble frameworks: (i) simple average (SA) ensemble, (ii) weighted average (WA) ensemble, and (iii) stacking or meta-learning (MTL) ensemble. In SA ensemble, the ensemble prediction was obtained by assigning uniform weight to the predicted values generated by all models (for model-level ensemble) or all chromosomes (for chromosome-level ensemble). The WA ensemble prediction was computed as a weighted average of the predicted values from all component of models/chromosomes, where the weight assigned to each model or chromosome was proportional to the inverse of the variance. The weights were normalized such that the sum of all weights equal to one. In MTL approach, predictions from all 14 base learners (including Bayesian, BLUP, and ML models) were used as inputs to a meta-learner. Ridge regression was employed as the meta-learner to generate the final ensemble prediction. The comparisons were made both at the model-level and chromosome-level ensemble. The GP accuracy was evaluated using the PCC between observed and ensemble predicted phenotypic values.

## Results

### Analysis of phenotypic data

**Figure 3:**
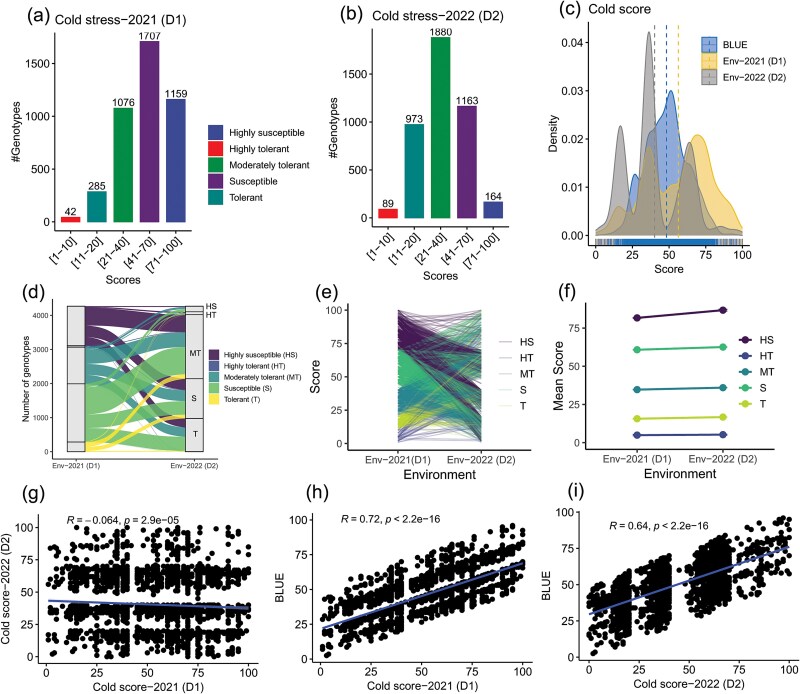
Distribution and relationship of cold stress tolerance scores across 2 years and best linear unbiased estimates (BLUE). (a, b) Frequency distribution of genotypes under cold stress conditions in 2021 (D1) and 2022 (D2). (c) Density plot showing the distribution of cold stress scores for D1, D2, and BLUE datasets. (d) Sankey diagram illustrating the transitions of genotypes among tolerance categories between D1 and D2. (e, f) Line plots showing individual and mean score changes across environments for different tolerance groups. (g–i) Scatter plots depicting correlations between cold scores of D1 and D2, D1 and BLUE, and D2 and BLUE datasets, respectively.

Out of 4269 genotypes, most of them were found to be either susceptible or moderately tolerant in both years (Fig. 3a and b). The year 2022 showed a little shift towards higher tolerance, as indicated by the increased number of tolerant, moderately tolerant, and highly tolerant genotypes and decreased number of susceptible and highly susceptible genotypes (Fig. 3a and b). Overall distribution of cold stress scores across environments and the BLUE values indicates that BLUE values lie intermediate to the two environments, reflecting effective adjustment for environmental variation (Fig. 3c). The Sankey plot shows how individual genotypes transitioned between tolerance categories from 2021 to 2022 (Fig. 3d). Specifically, most of the susceptible genotypes in 2021 become either moderately tolerant or tolerant in 2022. Similarly, around half of the susceptible genotypes in 2021 becomes either susceptible or tolerant in 2022 (Fig. 3d). On the other hand, some moderately tolerant and tolerant genotypes in 2021 becomes susceptible in 2022. This indicates the influence of the environment on the genotypes i.e., GE interaction. The lines connecting the scores of the genotypes in different environments indicate differential sensitivity to environmental changes (Fig. 3e) and the mean scores of all the genotypes increased slightly from 2021 to 2022 for most categories (Fig. 3f). A very weak negative correlation (R = −0.064, *P* = 2.9 × 10^−5^) between D1 and D2 scores indicates considerable environmental influence (Fig. 3g). In contrast, strong positive correlations between BLUE and individual environments (R = 0.72 with D1; R = 0.64 with D2) show that the BLUE estimates help effectively to minimizing environmental noise (Fig. 3h and i). Estimates of narrow-sense of heritability were observed 0.15, 0.13, and 0.19 for datasets D1, D2, and D3, respectively.

### Chromosome-wise genomic prediction accuracy

**Figure 4:**
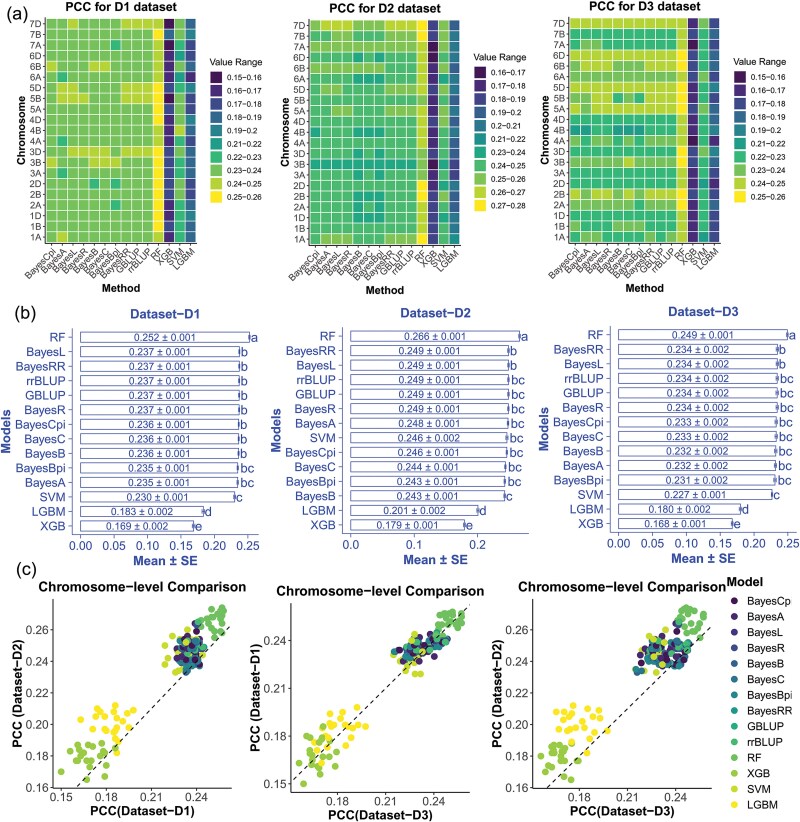
Chromosome-level genomic prediction accuracy across datasets and models. (a) Heat maps showing PCCs for chromosome-wise predictions across datasets using different genomic prediction models. (b) Comparison of estimates of PCC across models for each dataset, where different letters indicate statistically significant differences (*P* < .05). (c) Scatter plots showing comparison of chromosome-level PCC values between datasets, across all models.

For all three datasets, RF achieved higher accuracy than the other 13 models across all chromosomes (Fig. 4a and b). Except RF, the other three ML models such as SVM, XGB, and LGBM were observed to achieve less accuracy than that of Bayesian and BLUP models (Fig. 4a and b). The model-level scatterplots showed strong positive correlations, indicating that relative model rankings are same across datasets, but with small shifts in PCC values between datasets (Fig. 4c). For D1 and D3 datasets, the range of PCC were observed between 0.15 and 0.26, whereas for D2 it was observed in the range 0.16–0.28 (Fig. 4c). While the PCC of the models were averaged over chromosomes, the RF achieved significantly higher accuracy (D1: 0.252 ± 0.001, D2: 0.266 ± 0.001, D3: 0.249 ± 0.001) than that of Bayesian, BLUP, and other ML models (Fig. 4b). The accuracy of XGB model was observed significantly less as compared to other considered models, followed by LGBM.

### Chromosomal-level integrated genomic prediction accuracy

**Figure 5:**
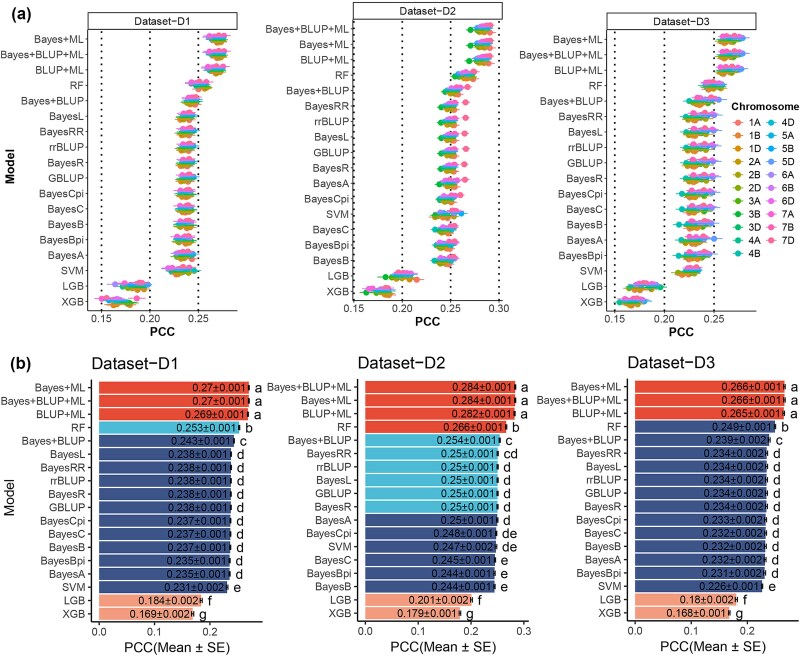
Genomic prediction accuracy of chromosomal-level integrated model. (a) PCCs for integrated and individual models for each chromosome. (b) Estimates of mean PCC for integrated and individual models, where different letters indicate statistically significant differences (*P* < .05).

The Bayesian, BLUP, and ML models were integrated for each chromosome. It was observed that integration of the ML models with Bayesian and BLUP models (Bayes+ML, Bayes+BLUP+ML, and BLUP+ML) yielded significantly higher GP accuracy (PCC) than that of individual models (Fig. 5a). Averaged across all chromosomes, Bayes+ML achieved mean PCC of 0.270 ± 0.001, 0.284 ± 0.001, and 0.266 ± 0.001 for D1, D2, and D3 datasets, respectively (Fig. 5b). Similarly, Bayes+BLUP+ML attained 0.270 ± 0.001, 0.284 ± 0.001, and 0.266 ± 0.001 across the three datasets. BLUP+ML also performed strongly with accuracies of 0.269 ± 0.001, 0.282 ± 0.001, and 0.265 ± 0.001, respectively (Fig. 5b). It was seen that RF consistently achieved higher PCC (D1: 0.253 ± 0.001, D2: 0.266 ± 0.001, D3: 0.249 ± 0.001) than that of integrated Bayesian+BLUP model (D1: 0.243 ± 0.001, D2: 0.254 ± 0.001, D3: 0.239 ± 0.002) (Fig. 5b). However, the ML models LGB and XGB achieved significantly less accuracy (PCC) not only than that of integrated models but also individual Bayesian and BLUP models (Tukey HSD test, *P* < .01). In respect of MSE, substantial variability was observed for the individual models but the integrated model showed more stable MSE across chromosomes (Supplementary Fig. 1a), indicating robustness of the integrated models. When averaged across chromosomes, the lowest MSE values were recorded for BLUP+ML (D1: 0.926 ± 0.001, D2: 0.934 ± 0.003, D3: 0.935 ± 0.002), Bayes+BLUP+ML (D1: 0.936 ± 0.003**,** D2: 0.954 ± 0.005, D3: 0.947 ± 0.003), GBLUP (D1: 0.937 ± 0.001, D2: 0.934 ± 0.001, D3: 0.944 ± 0.001), and RF (D1: 0.938 ± 0.001, D2: 0.932 ± 0.001, D3: 0.945 ± 0.002) (Supplementary Fig. 1b). Interestingly, the individual GBLUP and RF models produced the lowest MSE as compared to the integrated Bayes+ML model for all the three datasets.

### Model-level integrated genomic prediction accuracy

**Figure 6:**
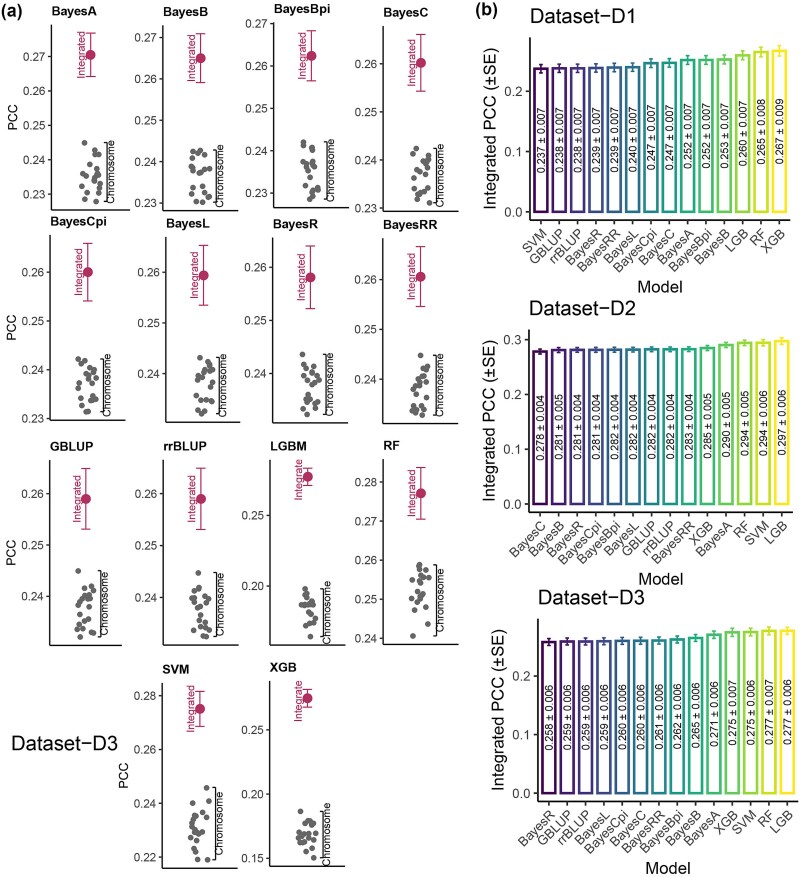
Genomic prediction accuracy of model-level integrated model. (a) PCCs for chromosome-wise and integrated genomic prediction model in dataset D3. (b) Estimates of mean integrated PCC across all models for datasets D1, D2, and D3.

For each model, we integrated the predicted phenotypic values obtained across chromosomes and computed the GP accuracy based on the integrated phenotypic values. It was observed that for each model, the GP accuracies (PCC) were increased after integrating chromosomal level prediction (Fig. 6a, Supplementary Figs 2a, 3, and  4). It was further observed that the ML models (LGB and XGB) that produced the least accuracy (PCC) while evaluated on individual chromosome (Fig. 4b), achieved the highest GP accuracy while integrating chromosomal-level prediction (Fig. 6b). The XGB model achieved the highest PCC for D1 dataset (0.267 ± 0.009), whereas LGB achieved the highest PCC for D2 (0.297 ± 0.006) and D3 (0.277 ± 0.006) datasets (Fig. 6b). The GBLUP and rrBLUP models were found achieving the lowest accuracy on the integrated datasets (Fig. 6b, Supplementary Figs 2b, 3, and  4). Though PCC values of the integrated model were observed higher than that of individual chromosome for all the GP models, the MSE values obtained on the integrated data were found less only for the ML models and GBLUP models (Supplementary Figs 2a, 3, and  4). Among all the models, the RF achieved the lowest MSE for all the three datasets (D1: 0.947 ± 0.010, D2: 0.912 ± 0.008, D3: 0.920 ± 0.009) (Supplementary Fig. 2b). It was also seen that the stability in PCC for across chromosomes are at par for all the models (Fig. 6b). However, with regard to MSE, the stability in accuracy was observed higher for the ML models and the GBLUP model as compared to the remaining GP models (Supplementary Fig. 2b).

### Analysis of convergence of fitness function

**Figure 7:**
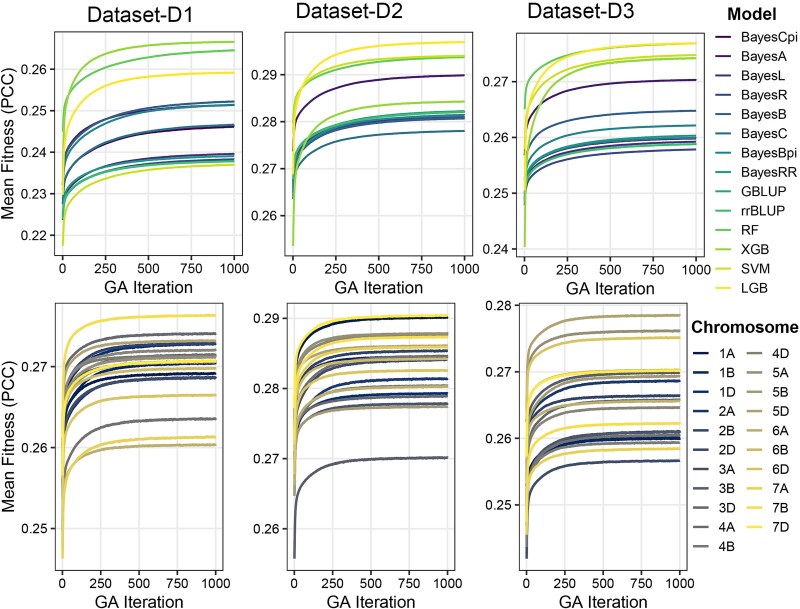
Convergence plots of the GA-based optimization procedure for model-level and chromosome-level ensemble integration across datasets D1, D2, and D3. The upper panel represents convergence of the GA optimization for integrating predictions from the 14 genomic prediction models, while the lower panel represents convergence during chromosome-level weight optimization across the 21 wheat chromosomes. The *x*-axis represents the number of GA iterations, and the *y*-axis represents the mean fitness value measured as the PCC between observed and integrated predicted phenotypic values. Across all datasets, the fitness values increased rapidly during the initial iterations and gradually approached a stable plateau, indicating effective convergence of the optimization process.

Weight optimization was performed independently within each fold of the five-fold cross-validation framework and repeated across all 100 repetitions. Since the composition of the training and testing sets changed across folds and repetitions, the optimized weights obtained through GA were also varied. Nonetheless, to assess the robustness of the GA optimization process, we evaluated the convergence behavior of the fitness function during the iterative search procedure. The fitness/objective function was defined as the maximization of the PCC between the observed phenotypic values and the ensemble-predicted phenotypic values within the optimization framework. During successive GA iterations, the fitness values increased initially and gradually stabilized, indicating convergence of the optimization process (Fig. 7). The convergence plots in Fig. 7 showed that the fitness values reached a stable plateau at ~1000 iterations, suggesting that the GA converged toward an optimal or near-optimal solution.

### Genomic prediction accuracy using CFMS and MFCS approaches

**Figure 8:**
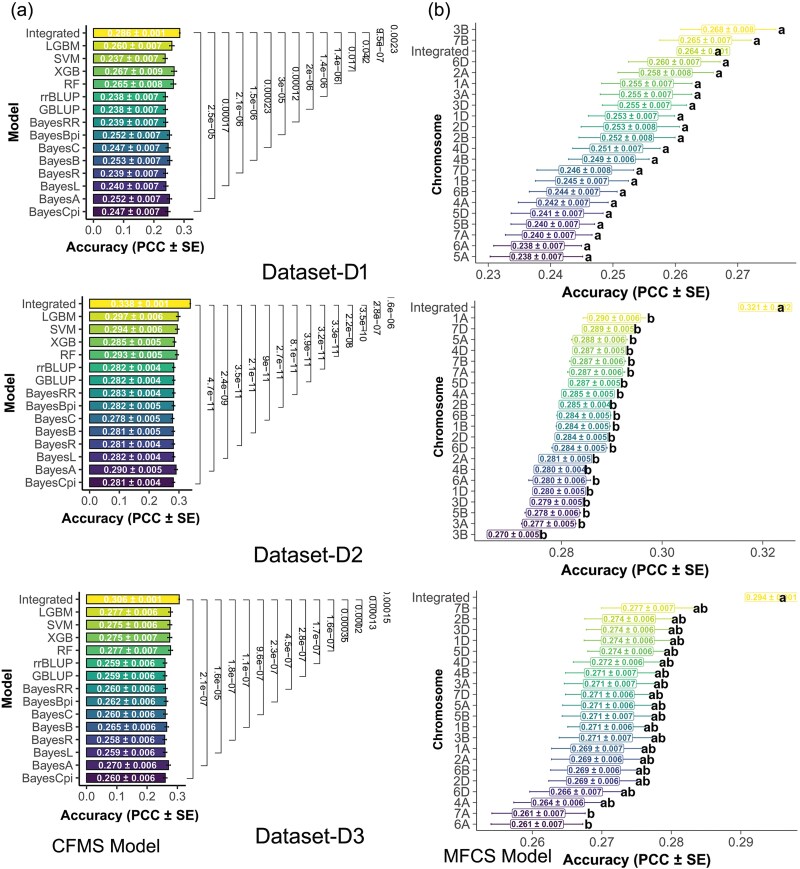
Genomic prediction accuracy of CFMS and MFCS integrated models across datasets. (a) Estimates of genomic prediction accuracy of different chromosomal-level genomic prediction models and CFMS integrated model for datasets D1, D2, and D3. (b) Estimates of genomic prediction accuracy of different chromosome-level and MFCS integrated model across datasets, with different letters indicating statistically significant differences (*P* < .05).

In all the three datasets, CFMS model achieved higher GP accuracies than that of MFCS model of integration (Fig. 8a and b). In addition, the integrated GP accuracy (PCC) for CFMS model was significantly higher as compared to the accuracy of the individual models (Fig. 8a). However, integrative accuracy of the MFCS model was significantly higher than that of individual chromosome for D2 and D3 datasets only (Fig. 8b). Specifically, the CFMS model achieved integrated accuracy (PCC) of 0.286 ± 0.001, 0.338 ± 0.001, 0.306 ± 0.001 for datasets D1, D2, and D3, respectively (Fig. 8a). The MFCS model achieved integrated accuracy (PCC) of 0.264 ± 0.001, 0.321 ± 0.002, 0.294 ± 0.001 for datasets D1, D2, and D3, respectively (Fig. 8b). Furthermore, the integrated MSE of CFMS model (0.922 ± 0.001) was found to be significantly less than that of individual model for dataset D1, whereas for dataset D2, the integrated MSE (0.943 ± 0.006) was found at par with that of ML models and significantly less than that of Bayesian and BLUP alphabets (Supplementary Fig. 5). In case of D3 dataset, the integrated MSE (0.988 ± 0.003) was found significantly higher than most of the individual models. This may be due to the fact that objective function of the GA in the present study was maximizing the PCC between observed and integrated predicted phenotypic values, rather than minimizing the MSE. Since PCC measures the strength and direction of the linear association between predicted and observed values, a model can achieve a high PCC even when the absolute prediction errors remain comparatively larger. In contrast, MSE is directly influenced by the magnitude of prediction errors and is therefore more sensitive to scale differences and extreme residual values. Therefore, improvement in PCC does not necessarily guarantee a proportional reduction in MSE. As far as integrated MSE for MFCS model is concerned, it was not found to be significantly less (D1: 0.943 ± 0.001, D2: 0.963 ± 0.003, 0.979 ± 0.002) as compared to individual chromosome-level integrated model (Supplementary Fig. 5). In terms of integrated MSE, the GP accuracy of CFMS model was found higher than that of MFCS for D1 and D2 datasets but not for D3 dataset (Supplementary Fig. 5).

### Comparative analysis with whole genome marker data

**Figure 9:**
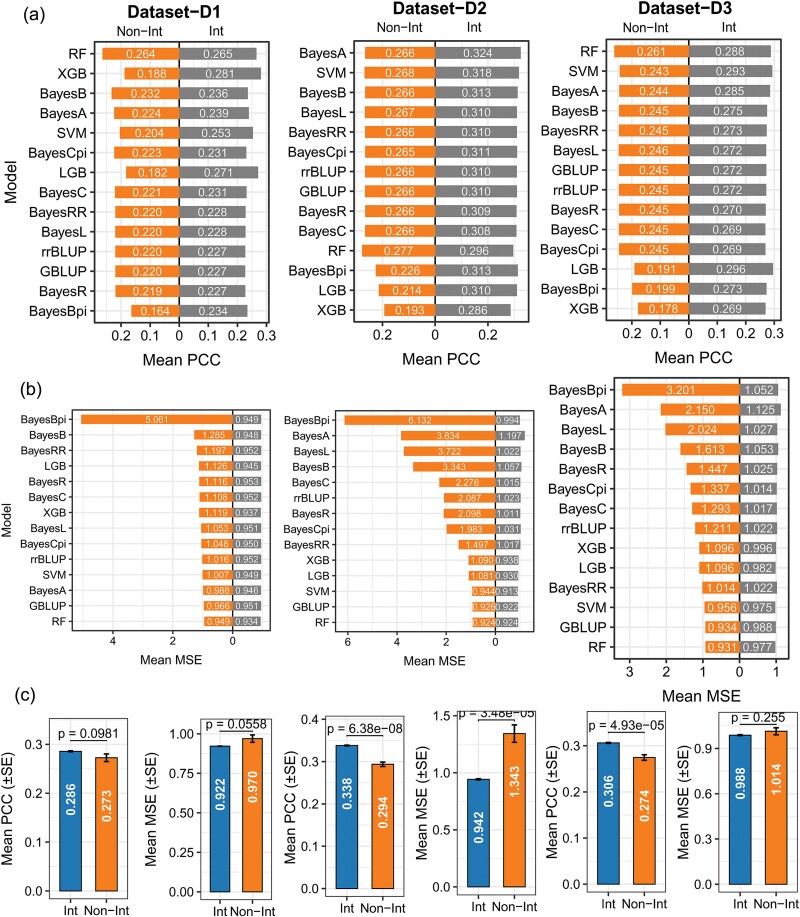
Comparison between chromosomal-partitioning integrated models and whole-genome (non-integrated) models. (a) PCC for non-integrated (whole-genome) and integrated (chromosomal-partitioning) models across datasets D1, D2, and D3. (b) MSE comparison between the two approaches across datasets. (c) Overall comparison showing the integration results of chromosomal-partitioning integrated models versus models trained on the whole-genome dataset, based on estimates of PCC and mean MSE, with corresponding *P*-values indicating statistical significance.

Across all datasets, the chromosome-integrated (Int) models (where predictions were first made for individual chromosomes and then integrated for each model) consistently achieved higher or comparable PCC values as compared to their non-integrated (Non-Int) counterparts (where prediction for each model was done on the entire genomic dataset without chromosomal partitioning) (Fig. 9a). The improvement was particularly higher for models such as XGB, LGB, BayesB, and SVM. For dataset D1, the improvement in PCC ranged from ~0.37% to 49%, with the highest gain observed for XGB (0.188 → 0.281). In dataset D2, PCC increased by about 8%–46%, with the highest improvement for LGB (0.214 → 0.310) and for dataset D3, PCC improved by 9%–50%, with the highest improvement for LGB (0.191 → 0.296) (Fig. 9a). The lowest improvement in accuracy across all datasets was observed for RF (0.264 → 0.265 in D1; 0.277 → 0.296 in D2; 0.261 → 0.288 in D3) (Fig. 9a). In terms of MSE, the GP accuracies were found higher for most of the models, barring exceptions (Fig. 9b). The highest reduction in MSE was observed for BayesBpi (from 5.061 → 0.949 in D1, 6.132 → 0.994 in D2, and 3.201 → 1.052 in D3) and lowest reduction in MSE was observed with RF for D1 (0.949 → 0.934) and D2 (0.924 → 0.924), whereas in D3, a marginal increase in MSE was noted for SVM, RF, GBLUP, and BayesRR models. At the model-level integration, the chromosomally integrated models consistently achieved higher mean PCC values (0.286 for D1, 0.338 for D2, and 0.306 for D3) compared with integrated models trained on the entire genomic dataset without chromosomal partitioning (0.273, 0.294, and 0.274, respectively) (Fig. 9c). Also, the MSE values for the chromosomally integrated models (0.922, 0.942, and 0.988) were lower than those of the non-integrated models (0.970, 1.343, and 1.014) (Fig. 9c).

### Comparative analysis with existing ensemble approaches

**Figure 10:**
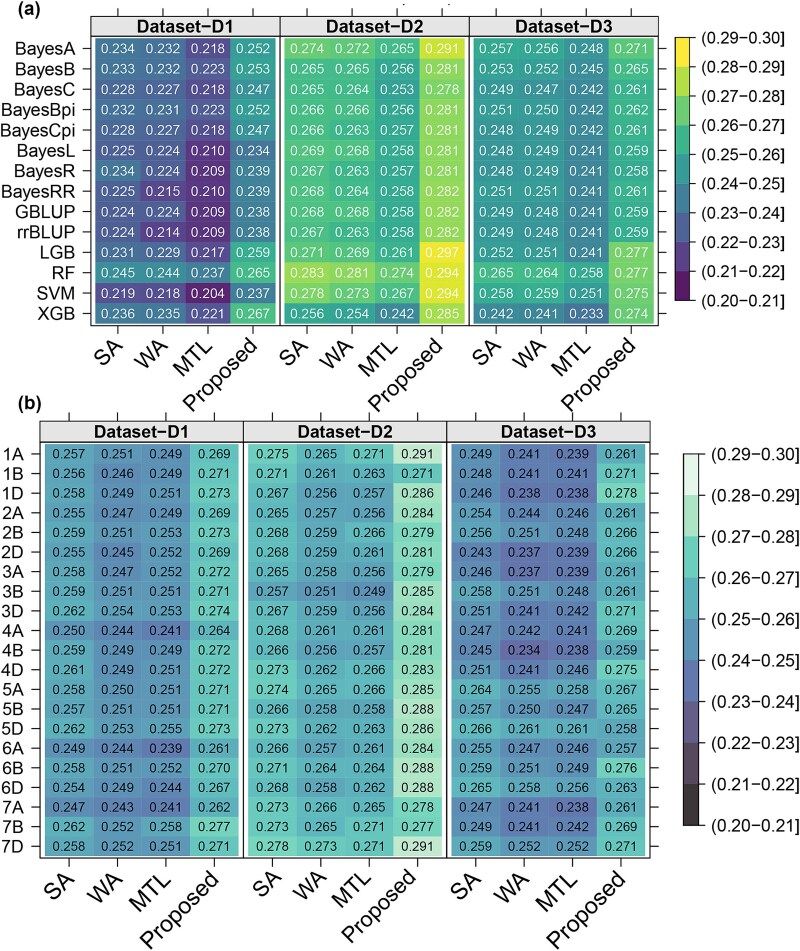
Comparative heatmap showing GP accuracies of the proposed ensemble framework and existing ensemble approaches. (a) Heatmap representing model-level ensemble GP accuracies for 14 genomic prediction models under SA, WA, MTL, and the proposed ensemble framework. (b) Heatmap representing chromosome-level ensemble GP accuracies across the 21 wheat chromosomes (1A–7D) using the same ensemble approaches. The numerical values within each cell indicate the PCC between observed and predicted phenotypic values.

The proposed ensemble framework achieved higher GP accuracy than the SA, WA, and MTL ensemble approaches at both chromosomal-level and model-level integration (Fig. 10). At the chromosomal-level ensemble, the proposed framework achieved the highest GP accuracy of 0.265 for dataset D1 using the RF model, whereas the highest accuracies achieved by the SA, WA, and MTL approaches were 0.245, 0.244, and 0.237, respectively, also with the RF model (Fig. 10a). Similarly, for dataset D2, the proposed ensemble framework achieved the highest GP accuracy of 0.294, outperforming the SA (0.283), WA (0.281), and MTL (0.271) approaches, with the RF model again achieving the highest accuracy among all individual models (Fig. 10a). For dataset D3, the proposed ensemble framework produced the highest GP accuracy of 0.277 with the RF model, compared to 0.264, 0.251, and 0.258 obtained using the SA, WA, and MTL approaches, respectively (Fig. 10a). At the model-level ensemble, the proposed framework achieved higher GP accuracies across chromosomes compared to the alternative ensemble approaches (Fig. 10b). For dataset D1, the GP accuracies obtained using the SA, WA, MTL, and proposed ensemble frameworks ranged from 0.250–0.262, 0.244–0.253, 0.241–0.258, and 0.262–0.277, respectively. Similarly, for dataset D2, the GP accuracies across chromosomes were observed in the ranges of 0.257–0.278 for SA, 0.251–0.273 for WA, 0.249–0.271 for MTL, and 0.277–0.288 for the proposed ensemble framework (Fig. 10b). For dataset D3, the chromosomal-level ensemble GP accuracies obtained using the SA, WA, MTL, and proposed ensemble approaches ranged from 0.243–0.265, 0.237–0.261, 0.238–0.261, and 0.257–0.278, respectively (Fig. 10b).

### System configuration and run time

The GP using 14 models across 21 chromosomes were performed on a Linux-based high-performance computing server equipped with dual AMD EPYC 7282 16-Core processors (32 physical cores and 64 threads) and 256 GB RAM, whereas the GA-based weight optimization was conducted on an iMac system equipped with an Apple M4 chip (10-core CPU comprising 4 performance cores and 6 efficiency cores) and 32 GB unified memory running macOS. Under the repeated five-fold cross-validation scheme with 100 iterations, fitting of 14 GP models across all 21 chromosomes required ~36 hours per iteration on average, whereas the GA-based optimization procedure required ~45 minutes per iteration for assigning optimized weights to chromosomes and models. Importantly, the majority of the computational time was associated with fitting the individual GP models across chromosomes rather than with the GA-based optimization. It is also important to note that the computational requirement of the proposed framework largely depends on the dimensionality of the dataset, including the number of genotypes, number of chromosomes, markers per chromosome, and the GP models employed. In the present study, the dataset consisted of 4269 phenotypic observations and 34 134 molecular markers employed, which substantially increased the computational time associated with model fitting and repeated cross-validation analyses.

## Discussion

In this study, we developed a sequential bidirectional integration strategy for improving the GP accuracy and employed it on cold stress tolerance trait in wheat. We utilized three datasets D1, D2, and D3, where the datasets D1 and D2 represent two environments from 2021 and 2022 winter sowing seasons and D3 represents the BLUE values of genotypes obtained after adjusting the environmental effect. The phenotypic values calculated as cold tolerance scores differed across the 2 years and found to be less correlated, highlighting the influence of environmental factors on phenotypic variation, as reported earlier in wheat and other crops [60–62]. Conversely, the strong correlation between BLUE and environment-specific data indicated that linear mixed effect models effectively adjusted the environmental heterogeneity.

The predictions on chromosome-partitioned subset of markers revealed that RF achieved higher accuracy than other models, which implies the capability of RF to capture nonlinear and higher-order marker–marker interactions [63, 64]. The relatively lower GP accuracy for XGB and LGBM compared to RF may be due to their dependence on gradient-based optimization, which may not fully capture complex additive and non-additive genetic effects. Bayesian and BLUP models demonstrated moderate accuracies, corroborates with previous findings that such models perform well when genetic architectures are largely additive [35, 65].

The GP accuracies were observed higher for chromosomes 2B, 3B, 4A, 5A, 5B, 5D, 6B, 6D, and 7D, suggesting that cold stress tolerance in wheat is a complex polygenic trait governed by both major-effect loci and multiple minor genes across the genome. The higher GP accuracy for the mentioned chromosomes also supports the existing studies. For instance, at least 10 chromosome pairs have been reported to be associated with cold stress tolerance, with strong evidence for group 5 chromosomes [66–70]. More recently, a major-effect QTL linked to frost tolerance was reported on chromosome 4A [71] and new cold-resistance genes such as *Wcr-3* and *Wcr-4* have been identified on chromosome 2B and 2D, respectively [72].

Integrating predictions of Bayesian, BLUP, and ML models for each chromosome improved the GP accuracy compared to individual models. The improved performance of integrated models (Bayes+ML and BLUP+ML) indicated the strong complementarity between parametric (Bayesian, BLUP) and nonparametric (ML) approaches [22, 73, 74], where ML algorithms capture nonlinear epistasis interactions effects and Bayesian/BLUP models capture additive genetic effects.

When predictions were integrated across the chromosomes at model-level, GP accuracy for each model increased. ML models such as XGB and LGB showed substantial improvements in GP accuracy, as compared to their chromosome-wise accuracy. This may be due to the appropriate weight assignment to different chromosomal-level prediction values. Improvement was less for BLUP models, may be due to the assumption of homogeneous marker effects and additive variance structures. These findings inferred that integrating chromosomal-level predictions can improve accuracy of the GP models, especially for nonlinear ML frameworks.

The proposed integrated GP approach used a bidirectional framework, where integrations were made across both chromosomes and models in two sequential manners, CFMS and MFCS. When for each model, predicted phenotypic values are integrated across all chromosomes, the variability introduced by chromosome-specific genetic architecture is averaged out. This resulted in more variability among the GP models and produced higher GP accuracy after integration at the model level. In other words, CFMS approach integrates chromosome-wise predictions first and leverages complementary predictive ability of Bayesian, BLUP, and ML methods, where the weight allocation by genetic algorithm was optimized across models that differed in bias-variance trade-offs. In case of MFCS approach, integrating models within a single chromosome first retained the chromosome-specific noise and subsequent integration across chromosome resulted in less GP accuracy. This suggests that CFMS is the better strategy for improving GP accuracy as compared to MFCS approach.

Many studies have explored the partitioning of genome-wide SNPs using various strategies to improve GP accuracy. Akdemir and Jannink [75] tried to capture local epistatic patterns by recursively dividing the genome into chromosomes and smaller contiguous marker regions. Separate local kernel matrices were constructed for each region to capture additive and local epistatic effects, and these regional kernels were then combined through a weighted multi-kernel model for GP, resulting in comparable accuracy and better explainability. Recently, Difabachew et al. [76] explored several haplotype-block methods for GP in wheat including fixed-length haplotypes (based on no. of SNPs or genetic distance) and linkage disequilibrium (LD)-based blocking. The results showed that partitioning into haplotype blocks improved GP accuracy for several oligogenic resistance traits by better capturing local epistatic effects and linkage patterns. Li et al. [77] proposed mbBayesABLD, a multibreed GP model based on LD block genomic partitioning, where chromosomes were divided into nonoverlapping LD blocks with block-specific marker variances. The study showed improved GP accuracy compared with conventional whole-genome models such as GBLUP and BayesR, particularly for small-population breeds. On the basis of significance of genomic partitioning in identifying the genetic regions contributing to particular traits and improving GP accuracy, we developed the chromosome-wise partition strategy that was integrated into an ensemble framework for GP.

A comparative analysis with genome-wide model revealed that chromosome-integrated models consistently achieved higher accuracy than the models trained on whole-genome data, demonstrating the effectiveness of chromosomal-partitioning for GP prediction. This improvement likely occurred because chromosomal integration reduces noise from genome-wide heterogeneity and enhances local linkage disequilibrium signals. Reduced MSE in integrated models across datasets further supported the generalization of the developed approach. These findings confirm that chromosome-level integration provides a biologically meaningful and statistically robust approach over traditional genome-wide approaches in GP. The developed ensemble framework also achieved higher GP accuracy compared with existing ensemble strategies for GP, which includes simple average, weighted average, and meta-learning based ensemble approaches.

**Key Points**

- Cold stress tolerance in wheat exhibits low heritability and complex genetic architecture, limiting the predictive performance of conventional genomic selection models.
- A dual, sequential genomic prediction framework integrating chromosome-wise and model-wise predictions was developed using a genetic algorithm.
- Both chromosome-first-model-second (CFMS) and model-first-chromosome-second (MFCS) strategies achieved higher prediction accuracy than individual models and whole-genome approaches.
- Chromosome-level integration enhanced biological relevance and reduced prediction error.

## Supplementary Material

Supplementary_File_bbag375

## Data Availability

The source code with step-by-step description of CFMS model is available at https://github.com/PrabinaMeher/OptimGS.git. The phenotypic and genotypic dataset would be made available upon reasonable request to the corresponding authors.
