# Supplementary material for: OptimGS: a dual integrative genomic prediction framework for improving cold stress tolerance in wheat
Source: Brief Bioinform. 2026 Jul 14;27(4):bbag375. doi: 10.1093/bib/bbag375 (PMC13367444; doi:10.1093/bib/bbag375)
Supplement: Supplementary_File_bbag375 [file supplementary_file_bbag375.docx]

**Brief description of genomic prediction models**

**Bayesian models**

The genomic prediction model can be formulated as a linear mixed-effect model, where the phenotypic value of the genotype is modelled as a sum of the overall mean, the genetic contribution from markers, and a residual error that is

$y_{i}=\mu+\sum_{j=1}^{p} z_{ij}u_{j}+\varepsilon_{i}$,

where, $y_{i}$ is the phenotypic trait value for the *i*^th^ genotype, $\mu$ is the overall mean, $z_{ij}$ is the genotypic value of the *j*^th^ marker for the ith genotype, $u_{j}$ is the random effect of *j*^th^ marker and $\varepsilon_{i}$ is the random residual error i.e., $\varepsilon_{i}\sim N(0, \sigma_{e}^{2})$. Bayesian approach deals with estimating posterior distribution of the marker effects ($u_{j}$) and the Bayesian alphabets mainly differ in assuming prior distribution of the marker effects and variance of the markers effect size. The assumptions for the prior distribution of the marker effects and their variances are shown in figure below (Figure S1).

**
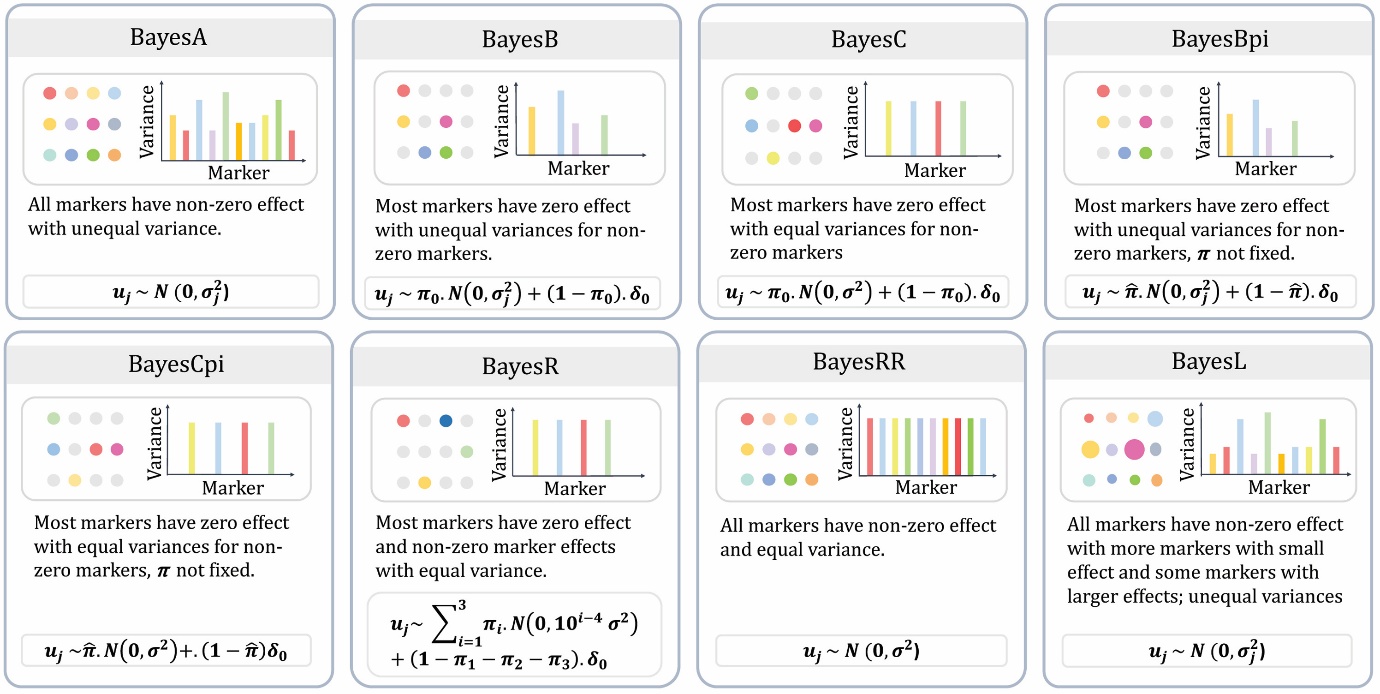
**

**Figure S1.** Schematic representation of various Bayesian genomic prediction models. Each colored dot represents a genetic marker, and the height of the bars indicates the variance of marker effects. Models differ in their assumptions about the proportion of markers with non-zero effects and the variance structure among these effects.

Except BayesL, the variance of the marker effects are assumed to follow inverse chi-square distribution i.e., $\sigma^{2}\sim\chi^{-2}(\nu,S)$ or $\sigma_{j}^{2}\sim\chi^{-2}(\nu,S)$, where $\nu$ is the degree of freedom with smaller value representing a larger marker effect and $S$ is the scale parameter with larger value indicates a higher probability of larger variance values and smaller value indicates smaller variance values. In case of BayesL, $\sigma_{j}^{2}\sim Exp(\lambda^{2}/2)$. The estimated proportion of marker effects $\hat{\pi}$ are assumed to follow Beta distribution i.e., $\hat{\pi}\sim Beta(\eta_{1},\eta_{2})$, where $\eta_{1}$ and $\eta_{2}$ are the shape parameters with $\eta_{1}>\eta_{2}$ denotes skewed distribution towards 1 indicating a prior belief that markers are more likely to have non-zero effects. If $\eta_{1}<\eta_{2}$ the distribution is skewed towards 0, indicating a prior belief that most of the markers have zero effects. If the marker effects are assumed to be mixture of more than two normal distribution, then $\hat{\pi}\sim Dirichlet(\eta)$, where $\eta$ is a positive integer.

**BLUP models**

The GBLUP model can be written as $\text{y}=\text{1}_{\mathbf{n}}\mu+\text{g}+\boldsymbol{\epsilon}$**,** where, $\text{y}$ is the $n$-dimensional vector of phenotypic records, $\mu$ denotes the overall mean, $\text{g}$ and $\boldsymbol{\epsilon}$ are the random vectors of additive genetic values and errors, respectively. Further, $\text{g}\sim N(\text{0},\text{G}\sigma_{g}^{2})$ and $\boldsymbol{\epsilon}\sim N(\text{0},\text{I}\sigma_{e}^{2})$, where $\text{G}$ is the genotypic relationship matrix (GRM). We computed the GRM using VanRaden approach (VanRaden 2008), that is, $\text{G}=\frac{\text{WW}^{T}}{2\sum_{j=1}^{m}p_{j}(1-p_{j})}$, where $w_{ij}=z_{ij}-2p_{j}$ and $p_{j}$ denotes the allelic frequency of the $j^{th}$ marker. For the GBLUP model, $\overset{\mathbf{^}}{\text{g}}\boldsymbol{=}{\boldsymbol{G(G+}\frac{\sigma_{e}^{2}}{\sigma_{g}^{2}}\boldsymbol{I)}}^{\boldsymbol{-}1}\boldsymbol{y}$ is the genomic estimated breeding value (GEBV). The rrBLUP model can be written as $\text{y}=\text{1}_{\mathbf{n}}\mu+\text{Z}\mathbf{u}+\boldsymbol{\epsilon}$**,** where $\boldsymbol{Z}$ is the marker genotype matrix, $\boldsymbol{u}$ is the vector of random marker effects and other notations are same as GBLUP model. The estimates of marker effect can be obtained as $\overset{\mathbf{^}}{\text{u}}\boldsymbol{=}{\boldsymbol{Z'(ZZ'+}\frac{\sigma_{e}^{2}}{\sigma_{u}^{2}}\boldsymbol{I)}}^{\boldsymbol{-}1}\boldsymbol{y}$**.**

**Machine learning models**

1. ***Support vector machine (SVM)***

- Find a function $f(x)=\langle w,\phi(x)\rangle+b$ that predicts $y$ with a margin of tolerance ($\epsilon$), where the weight vector w and the bias b are obtained by minimising the model complexity (${||w||}^{2}$) and error beyond margin ($\xi_{i},{\xi_{i}}^{*}$).
- Minimize the model complexity and error margin ($\xi_{i},{\xi_{i}}^{*}$) that is $Minimize:\frac{1}{2}{||w||}^{2}+C\sum_{i=1}^{n} (\xi_{i}+{\xi_{i}}^{*})$ subject to $y_{i}-f(x_{i})\leq\varepsilon+\xi_{i},f(x_{i})-y_{i}\leq\varepsilon+{\xi_{i}}^{*}$, where $\xi_{i},{\xi_{i}}^{*}$ are the slack variables representing deviations above and below the margin, $C$is the regularization parameter that controls the trade-off between model simplicity and error tolerance
- Transfer the primal to a dual optimization problem by using the langragian multipliers $\alpha_{i},{\alpha_{i}}^{*}\geq0$ that is $\max_{\alpha,\alpha^{*}} -\frac{1}{2}\sum_{i=1}^{n} \sum_{j=1}^{n} \left( \alpha_{i}-\alpha_{i}^{*} \right)\left( \alpha_{j}-\alpha_{j}^{*} \right)K\left( x_{i},x_{j} \right)+\sum_{i=1}^{n} \left( \alpha_{i}-\alpha_{i}^{*} \right)y_{i}-\varepsilon\sum_{i=1}^{n} \left( \alpha_{i}+\alpha_{i}^{*} \right)$ subject to $\sum_{i=1}^{n} (\alpha_{i}-\alpha_{i}^{*})=0$ and $0\leq\alpha_{i},{\alpha_{i}}^{*}\leq C,\forall i$, where *K*(.) represents the kernel function represented as the inner product $\langle\phi(x_{i}),\phi(x_{j})\rangle$ in the feature space.
- Use optimization techniques to solve the dual problem and find the optimal values of $\alpha_{i}$ and $\alpha_{i}^{*}$. Compute the weight vector $w$ using the optimal values of $\alpha_{i}$ and $\alpha_{i}^{*}$, $w=\sum_{i=1}^{n} \left( \alpha_{i}-\alpha_{i}^{*} \right)\phi(x_{i})$. Compute the bias $b$ using the support vectors (instances with $0\leq\alpha_{i},{\alpha_{i}}^{*}\leq C$), $b=y_{i}-\left\langle w,\phi\left( x_{i} \right) \right\rangle-\varepsilon$
- For a new genotype x, predict the phenotype $\overset{^}{y}$ using the equation $\overset{^}{y}=\sum_{i=1}^{n} \left( \alpha_{i}-\alpha_{i}^{*} \right)K\left( x_{i},x \right)+b$

1. ***Xtreme gradient boosting (XGBoost) and light gradient boosting (LightGBM)***

The steps involved in both XGBoost and LightGBM are largely similar. However, key differences arise in how splits and tree growth are handled. XGBoost evaluates all possible split points for each feature, whereas LightGBM accelerates this process by approximating splits through histogram**-**based binning of feature values. This reduces computational cost and memory usage. Moreover, LightGBM employs a leaf-wise tree growth strategy, where the leaf with the highest loss reduction (gain) is split first. In contrast, XGBoost follows a level-wise approach, expanding all leaves at the same depth simultaneously. The leaf-wise strategy in LightGBM allows deeper, more flexible trees that can capture complex patterns with higher accuracy, though it also increases the risk of over fitting. The steps are as follows:

- For each genotype $i$, compute the gradient $g_{i}=\frac{\partial L(y_{i},\hat{y}_{i})}{\partial\hat{y}_{i}}$ and Hessian $h_{i}=\frac{\partial^{2}L(y_{i},\hat{y_{i}})}{\partial\hat{y}_{i}2}$ of the loss function $L(y_{i},\hat{y}_{i})$ , where the initial prediction value ${\overset{̂}{\hat{y}}}_{0}=\frac{1}{n}\overset{n}{\underset{i=1}{\sum}}y_{i}$
- Split the data into leaves (subgroups) based on the feature values (e.g., genetic markers). For each leaf $j$, calculate the gradient $G_{j}=\underset{i\epsilon leaf_{j}}{\sum}g_{i}$ and Hessian $H_{j}=\underset{i\epsilon leaf_{j}}{\sum}h_{i}$
- Calculate the gain in the loss function $Gain=\frac{1}{2}\left( \frac{G_{L}^{2}}{H_{L}+\lambda}+\frac{G_{R}^{2}}{H_{R}+\lambda}-\frac{(G_{L}+G_{R})^{2}}{H_{L}+H_{R}-\lambda} \right)-\gamma$, after splitting the data into two leaves (left and right), where $G_{L}$ and $G_{R}$ are sum of the gradient of the left and right leaves after split, λ is the regularization term and $\gamma$ represents minimum loss reduction required to make a split.
- For each leaf $j$, compute the weight $w_{j}=-\frac{G_{j}}{H_{j}+\lambda}$
- Update the predictions for each genotype $i$ of the leaf $j$ by adding the leaf weight multiplied by a learning rate ($\nu$) i.e., $\hat{y}_{i}^{(t)}=\hat{y}_{i}^{(t-1)}+\nu w_{j}^{(t)}$
- Make the prediction for a test genotype $x_{test}$ after $T$ round of boosting $\hat{y}_{test}=\hat{y}_{0}+\nu\overset{T}{\underset{t=1}{\sum}}w_{t}$

In each round of boosting (1 to *T*), a new tree is added to the model, and the predictions are updated based on the residuals (errors) from the previous round.

1. ***Random forest (RF)***

- Create B bootstrap sample $D_{1},D_{2},...,D_{b}$from the training dataset D, where each bootstrap sample $D_{b}$ is created by randomly sampling N observations from D with replacement.
- Train a base regression model (regression tree) $f_{b}(x)$ on each bootstrap sample $D_{b}$. For each feature $j$ and split point $t$ (where a subset of features are selected to decide the splitting at each node), the best split is the one that minimizes the MSE tht is $\frac{1}{N}(\underset{i\in D_{l}}{\sum}(y_{i}-\overline{y}_{l})^{2}+\underset{i\in D_{r}}{\sum}(y_{i}-\overline{y}_{r})^{2})$, where $D_{l}$ and $D_{r}$ are the left and right subset after split.
- Repeat the split process recursively for $D_{l}$ and $D_{r}$ until stopping criterion is met (maximum depth or minimum sample per leaf).
- Make the prediction for a test instance (*x*), where the predicted value is the average of the of the target values of the observation in the falling leaf that is $\overset{̂}{\hat{y}}=\frac{1}{|D_{leaf}|}\underset{i\in D_{leaf}}{\sum}y_{i}$.

**Supplementary Figures**


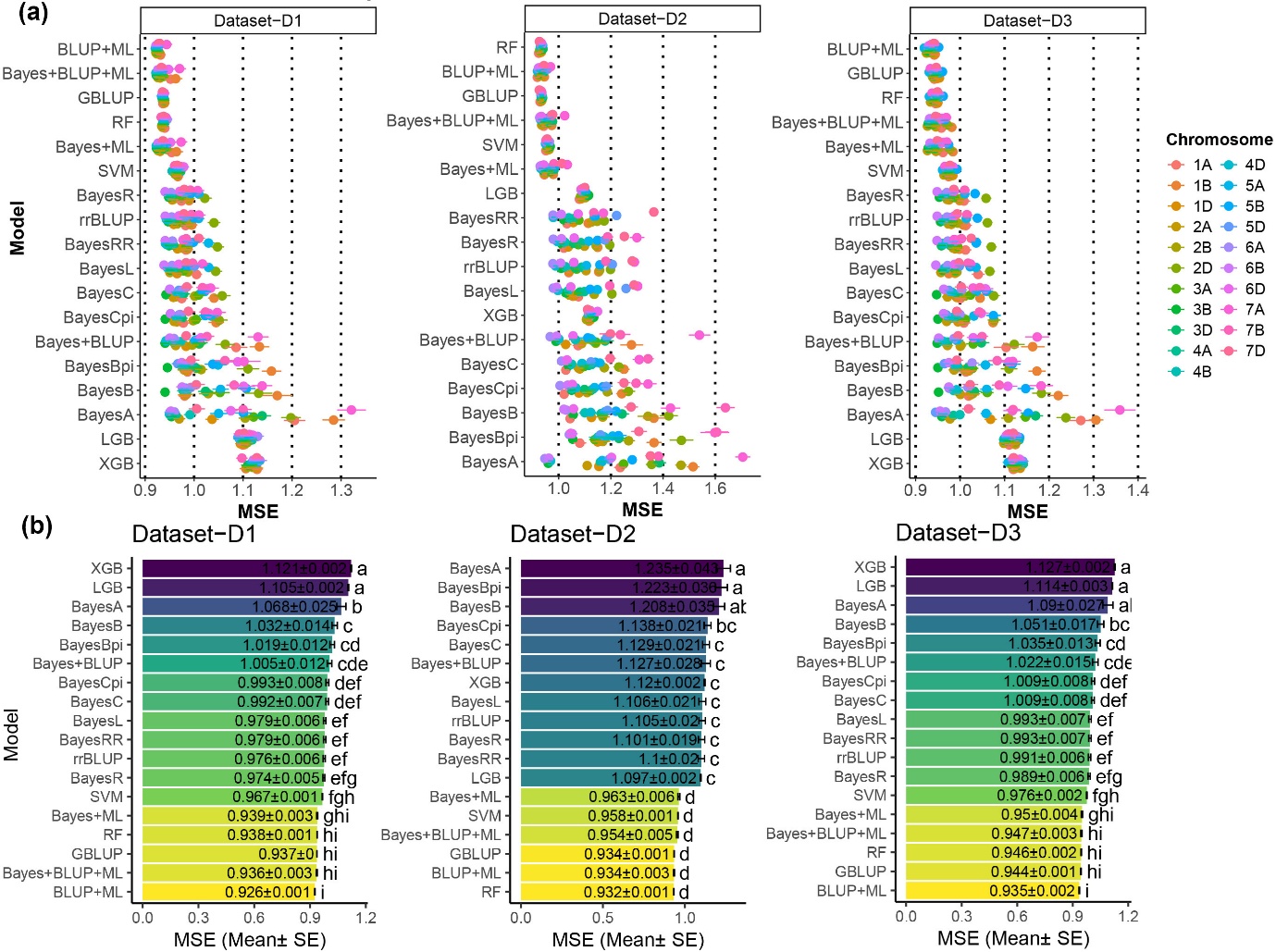


**Supplementary Figure 1.** Genomic prediction accuracy of chromosomal-level integrated model. (a) Chromosome-level mean squared error (MSE) for individual and integrated models across datasets. (b) Estimates of mean MSE for each model, where different letters denote statistically significant differences (p < 0.05).


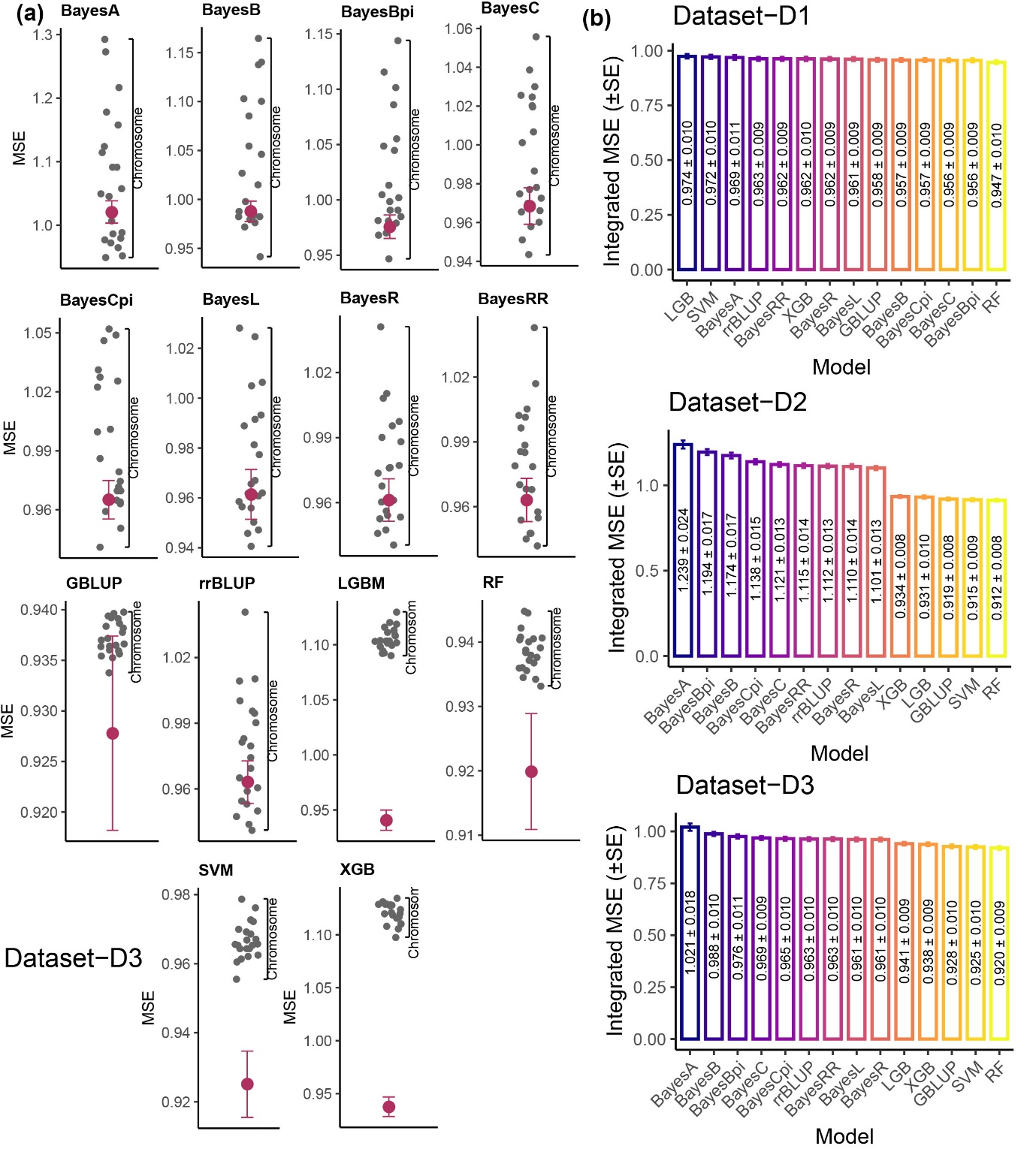


**Supplementary Figure 2.** Genomic prediction accuracy of model-level integrated model. (a) Chromosome-wise and integrated mean squared error (MSE) for each model in dataset D3. (b) Estimates of mean integrated MSE across models for datasets D1, D2 and D3.


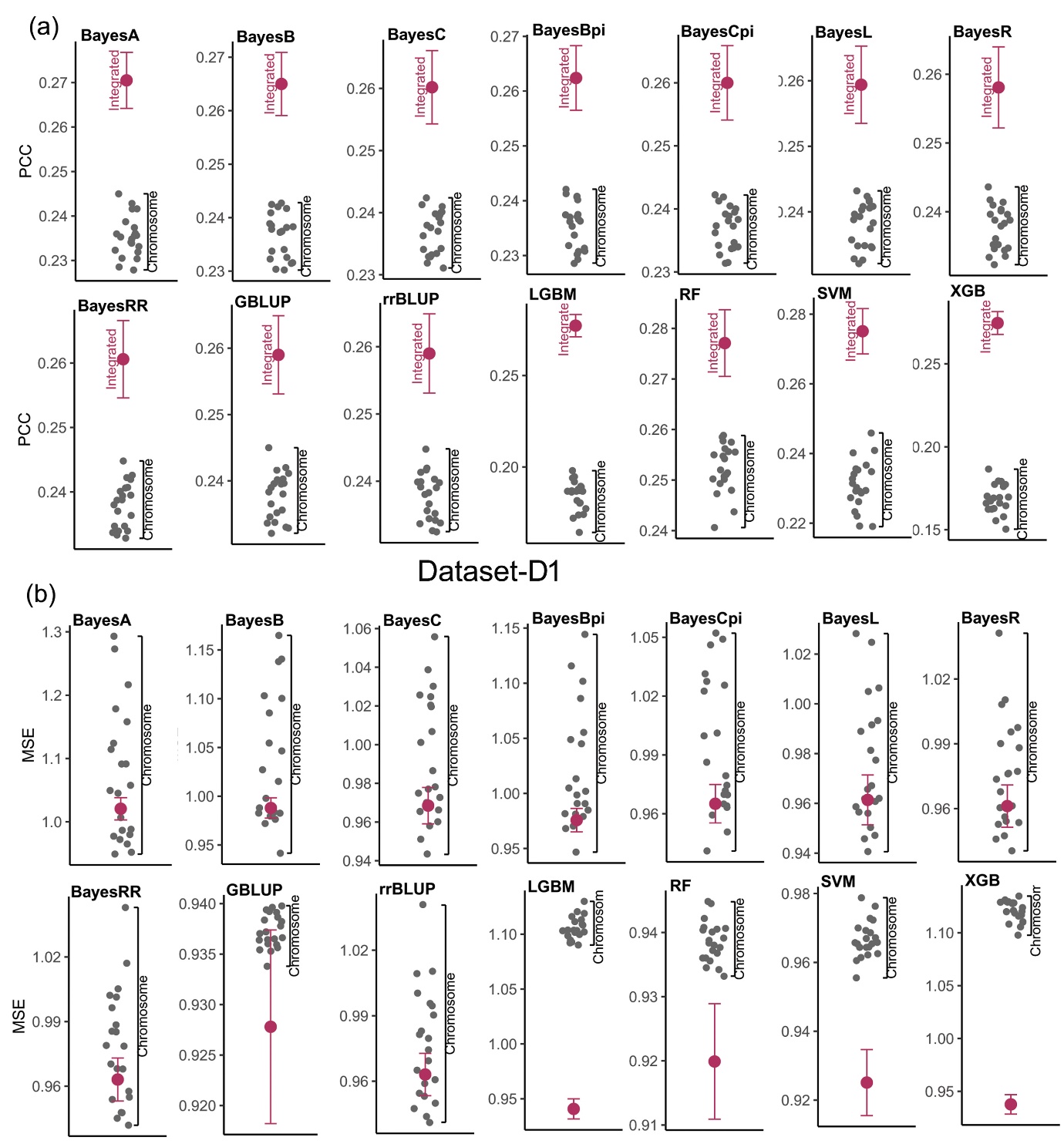


**Supplementary Figure 3.** Genomic prediction accuracy of model-level integrated model in D1 dataset. (a) Chromosome-wise and integrated genomic prediction accuracy (PCC) for each genomic prediction model. (b) Chromosome-wise and integrated mean squared error (MSE) for each model.

**
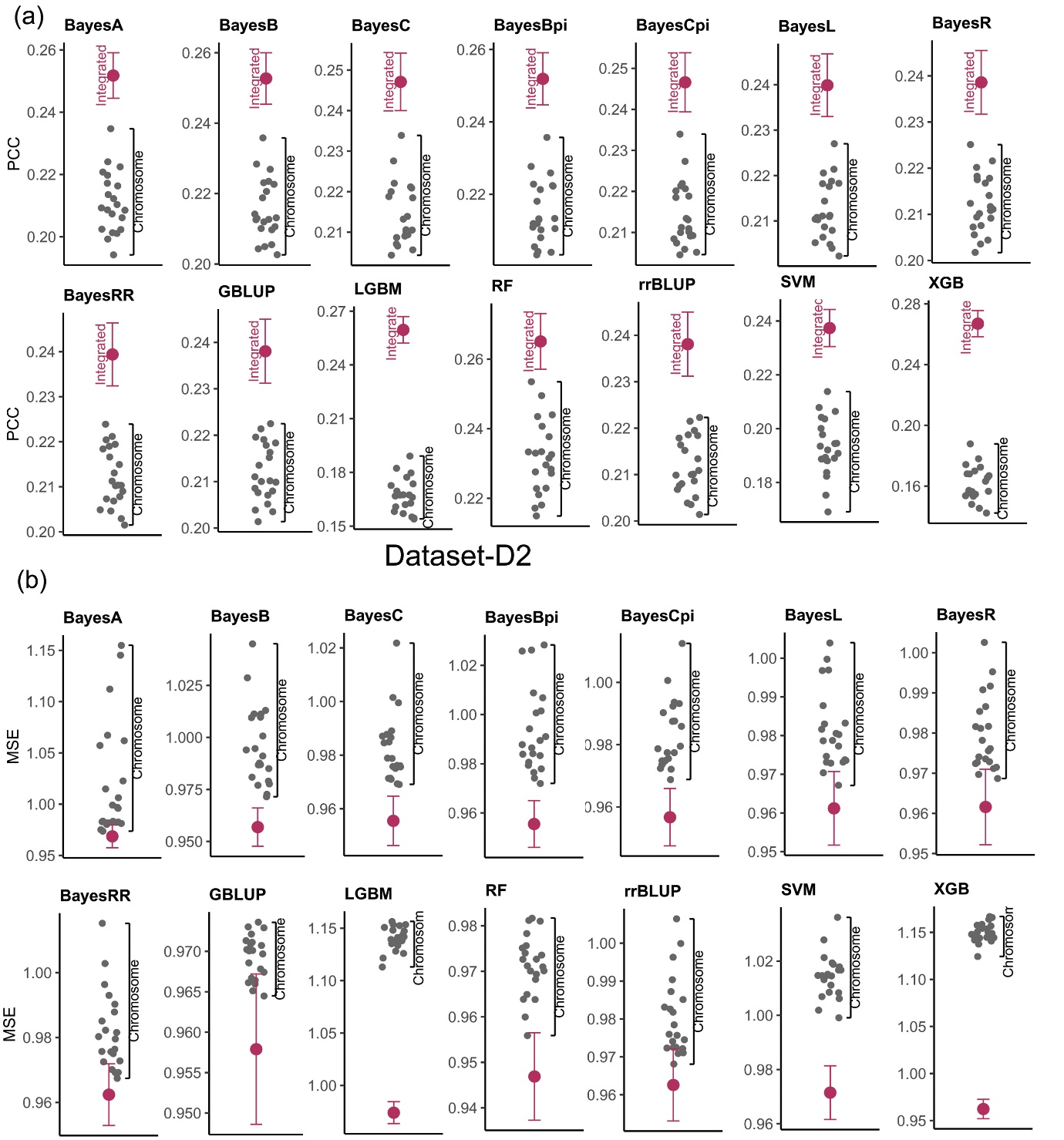
**

**Supplementary Figure 4.** Genomic prediction accuracy of model-level integrated model in D2 dataset. (a) Chromosome-wise and integrated genomic prediction accuracy (PCC) for each genomic prediction model. (b) Chromosome-wise and integrated mean squared error (MSE) for each model.

**
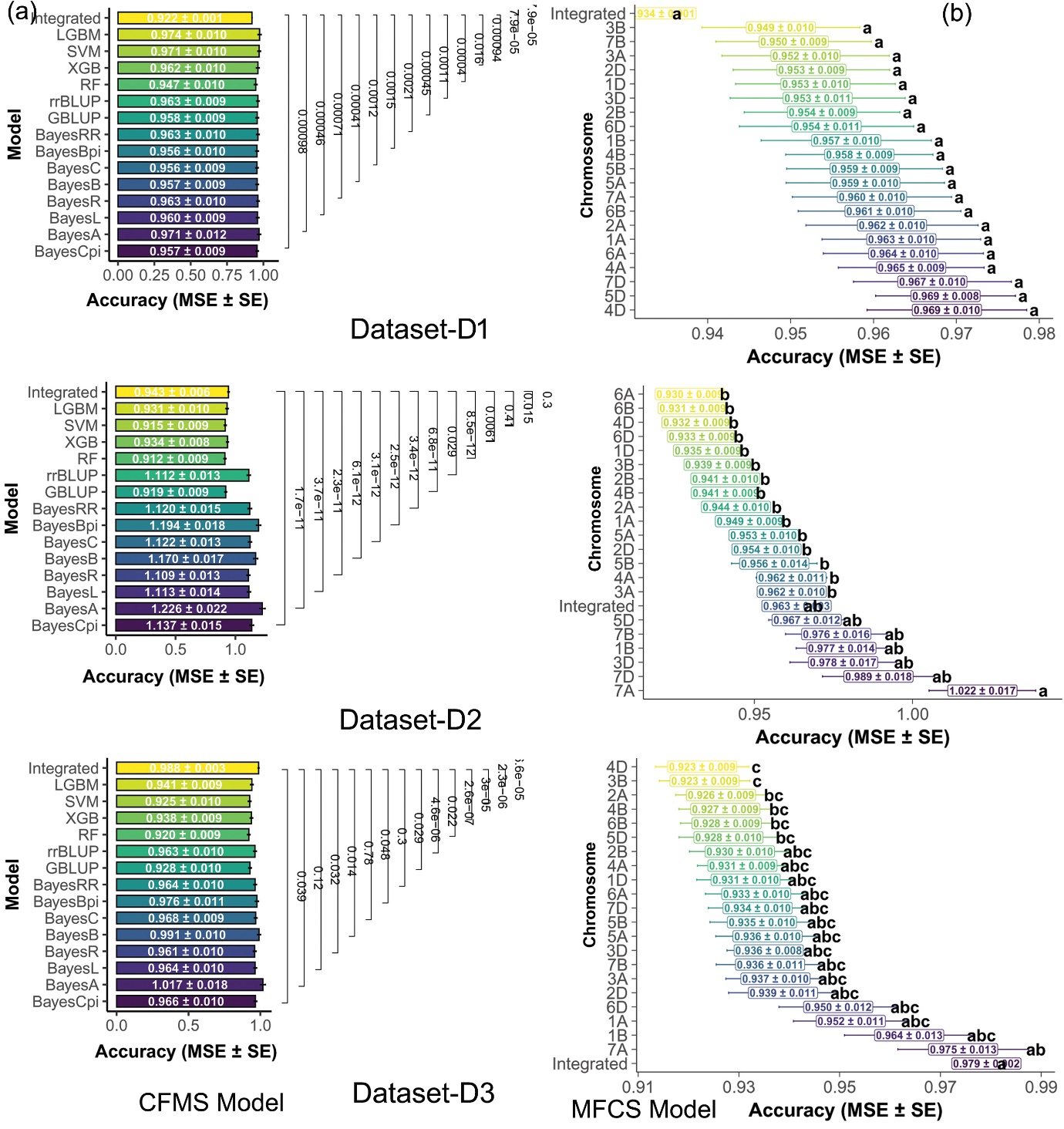
**

**Supplementary Figure 5.** Genomic prediction accuracy (MSE) of CFMS and MFCS integrated models across datasets. (a) Estimates of MSE of different chromosomal-level genomic prediction models and CFMS integrated model for datasets D1, D2, and D3. (b) Estimates of MSE of different chromosome-level and MFCS integrated model across datasets, with different letters indicating statistically significant differences (p < 0.05).
